# Microbial Plankton Community Structure and Function Responses to Vitamin B_12_ and B_1_ Amendments in an Upwelling System

**DOI:** 10.1128/AEM.01525-21

**Published:** 2021-10-28

**Authors:** Vanessa Joglar, Benjamin Pontiller, Sandra Martínez-García, Antonio Fuentes-Lema, María Pérez-Lorenzo, Daniel Lundin, Jarone Pinhassi, Emilio Fernández, Eva Teira

**Affiliations:** a Centro de Investigación Mariña da Universidade de Vigo, Departamento de Ecología y Biología Animal, Universidade de Vigo, Vigo, Spain; b Centre for Ecology and Evolution in Microbial Model Systems, Linnaeus University, Kalmar, Sweden; Norwegian University of Life Sciences

**Keywords:** vitamin B_12_, vitamin B_1_, community composition, nutrient limitation, cobalamin, thiamine, metatranscriptomics

## Abstract

B vitamins are essential cofactors for practically all living organisms on Earth and are produced by a selection of microorganisms. An imbalance between high demand and limited production, in concert with abiotic processes, may explain the low availability of these vitamins in marine systems. Natural microbial communities from surface shelf water in the productive area off northwestern Spain were enclosed in mesocosms in winter, spring, and summer 2016. In order to explore the impact of B-vitamin availability on microbial community composition (16S and 18S rRNA gene sequence analysis) and bacterial function (metatranscriptomics analysis) in different seasons, enrichment experiments were conducted with seawater from the mesocosms. Our findings revealed that significant increases in phytoplankton or prokaryote biomass associated with vitamin B_12_ and/or B_1_ amendments were not accompanied by significant changes in community composition, suggesting that most of the microbial taxa benefited from the external B-vitamin supply. Metatranscriptome analysis suggested that many bacteria were potential consumers of vitamins B_12_ and B_1_, although the relative abundance of reads related to synthesis was ca. 3.6-fold higher than that related to uptake. *Alteromonadales* and *Oceanospirillales* accounted for important portions of vitamin B_1_ and B_12_ synthesis gene transcription, despite accounting for only minor portions of the bacterial community. *Flavobacteriales* appeared to be involved mostly in vitamin B_12_ and B_1_ uptake, and *Pelagibacterales* expressed genes involved in vitamin B_1_ uptake. Interestingly, the relative expression of vitamin B_12_ and B_1_ synthesis genes among bacteria strongly increased upon inorganic nutrient amendment. Collectively, these findings suggest that upwelling events intermittently occurring during spring and summer in productive ecosystems may ensure an adequate production of these cofactors to sustain high levels of phytoplankton growth and biomass.

**IMPORTANCE** B vitamins are essential growth factors for practically all living organisms on Earth that are produced by a selection of microorganisms. An imbalance between high demand and limited production may explain the low concentration of these compounds in marine systems. In order to explore the impact of B-vitamin availability on bacteria and algae in the coastal waters off northwestern Spain, six experiments were conducted with natural surface water enclosed in winter, spring, and summer. Our findings revealed that increases in phytoplankton or bacterial growth associated with B_12_ and/or B_1_ amendments were not accompanied by significant changes in community composition, suggesting that most microorganisms benefited from the B-vitamin supply. Our analyses confirmed the role of many bacteria as consumers of vitamins B_12_ and B_1_, although the relative abundance of genes related to synthesis was ca. 3.6-fold higher than that related to uptake. Interestingly, prokaryote expression of B_12_ and B_1_ synthesis genes strongly increased when inorganic nutrients were added. Collectively, these findings suggest that upwelling of cold and nutrient-rich waters occurring during spring and summer in this coastal area may ensure an adequate production of B vitamins to sustain high levels of algae growth and biomass.

## INTRODUCTION

B vitamins are important growth factors with central roles in several metabolic pathways (1–3). In particular, B_12_ and B_1_ are essential cofactors in the primary metabolism of almost all forms of life (1, 4–6). Exogenous B-vitamin requirements seem to be widespread in marine ecosystems. This is relevant for coastal zone management, since almost all harmful algal bloom (HAB) species are auxotrophic for B_12_ and three-fourths require B_1_ (7). Marine prokaryotes are the ultimate source of exogenous B_12_ for marine phytoplankton, as eukaryotes lack the pathway for *de novo* synthesis of B_12_ (8). *De novo* synthesis of B_1_ is also limited to certain taxa, making most microbial plankton species dependent on an external supply (4, 9–11). In addition to being the source of B_12_ (12–14), prokaryotes often play an important ecological role as B-vitamin consumers (5, 15).

A considerable number of studies have demonstrated that B-vitamin auxotrophy is widespread in marine systems, with a vast number of marine microbial species requiring the uptake of at least one B vitamin (13, 16–21). In coastal systems, availability of B vitamins affects phytoplankton community size structure (15, 22) and shifts phytoplankton community composition from auxotrophs to nonauxotrophs when vitamin levels are reduced (15, 23). Experimental amendments of B_12_ and/or B_1_ often enhance phytoplankton biomass (13, 22, 24, 25). Some studies show that picoplankton appears to be responsible for the majority of B-vitamin uptake (15, 22). However, most B-vitamin amendment experiments with natural microbial communities lack detailed analyses of changes in taxonomic composition and, to the best of our knowledge, do not address functional changes at the level of genes in response to vitamin enrichment.

The coastal waters off northwestern Spain are characterized by the dominance of dinoflagellates, diatoms, or chlorophytes (25–27), that include many species described as auxotrophic for B_12_ (8, 10, 21, 28). The prokaryote community in this area is dominated by *Flavobacteriaceae* taxa (29, 30) that are mostly recognized as B_12_ auxotrophs (19). However, prokaryote taxa including B_12_ prototrophs, such as *Rhodobacterales* (19), *Synechococcus* (21, 31), and *Thaumarchaeota* (32), are also abundant (25, 27, 33). B_1_ auxotrophs, such as many dinoflagellates (4, 5, 18), often form important blooms in this area (25–27). Regardless of the abundance of potential B_12_ auxotrophs, field measurements in the northwestern coast of Spain reported very low B_12_ concentrations (<2.7 pM) along a cross-shelf transect, with higher values near the coast during spring and summer (27, 34). Moreover, a set of short-term B_12_ and/or B_1_ amendment experiments in this area revealed a relatively reduced and temporally variable response in terms of chlorophyll *a* (Chl-*a*) or prokaryote biomass to the input of these vitamins (25). The results from these experiments, conducted to specifically explore the short-term, seasonal, and spatial variability of the response of prokaryotes and phytoplankton in terms of biomass, suggested that the microbial plankton community in this area could be well adapted to cope with B-vitamin shortage and that a close balance exists between production and consumption of these important growth factors (25).

As detailed above, we recently intensified our study of how vitamin dynamics influences the planktonic system in the productive Atlantic waters off the northwestern Iberian Peninsula (25, 27). However, changes in microbial taxonomic composition or gene expression associated with vitamin B_12_ and/or B_1_ additions were not investigated in these studies. In order to gain insight into the compositional and functional microbial plankton responses to B_12_ and/or B_1_ amendments in this productive region, here we conducted an additional set of experiments to assess changes in microbial community composition as well as changes in the prokaryote expression of B_12_ and B_1_ genes.

Within this context, we hypothesized that vitamin B_12_ and B_1_ supply can force changes in the composition and function of the microbial community. Enrichment microcosm experiments were conducted using natural communities from surface water collected at a shelf station off northwestern Spain in winter, spring, and summer to explore changes in prokaryote and eukaryote community taxonomic composition. Moreover, changes in prokaryotic gene expression after vitamin B_12_ and/or B_1_ additions in one experiment conducted in February were analyzed. Our findings suggest that although most prokaryotes were capable of synthesizing vitamins B_12_ and/or B_1_, some taxa appeared to be mostly consumers of these cofactors. The combination of metatranscriptomics analyses and B vitamin amendment experiments with natural plankton communities allowed the identification of the metabolically active prokaryotes and their role as vitamin producers and/or consumers in this marine costal area, which is key to better understanding the processes sustaining the ecosystem functioning.

## RESULTS

### Microbial dynamics in the mesocosms.

**FIG 1:**
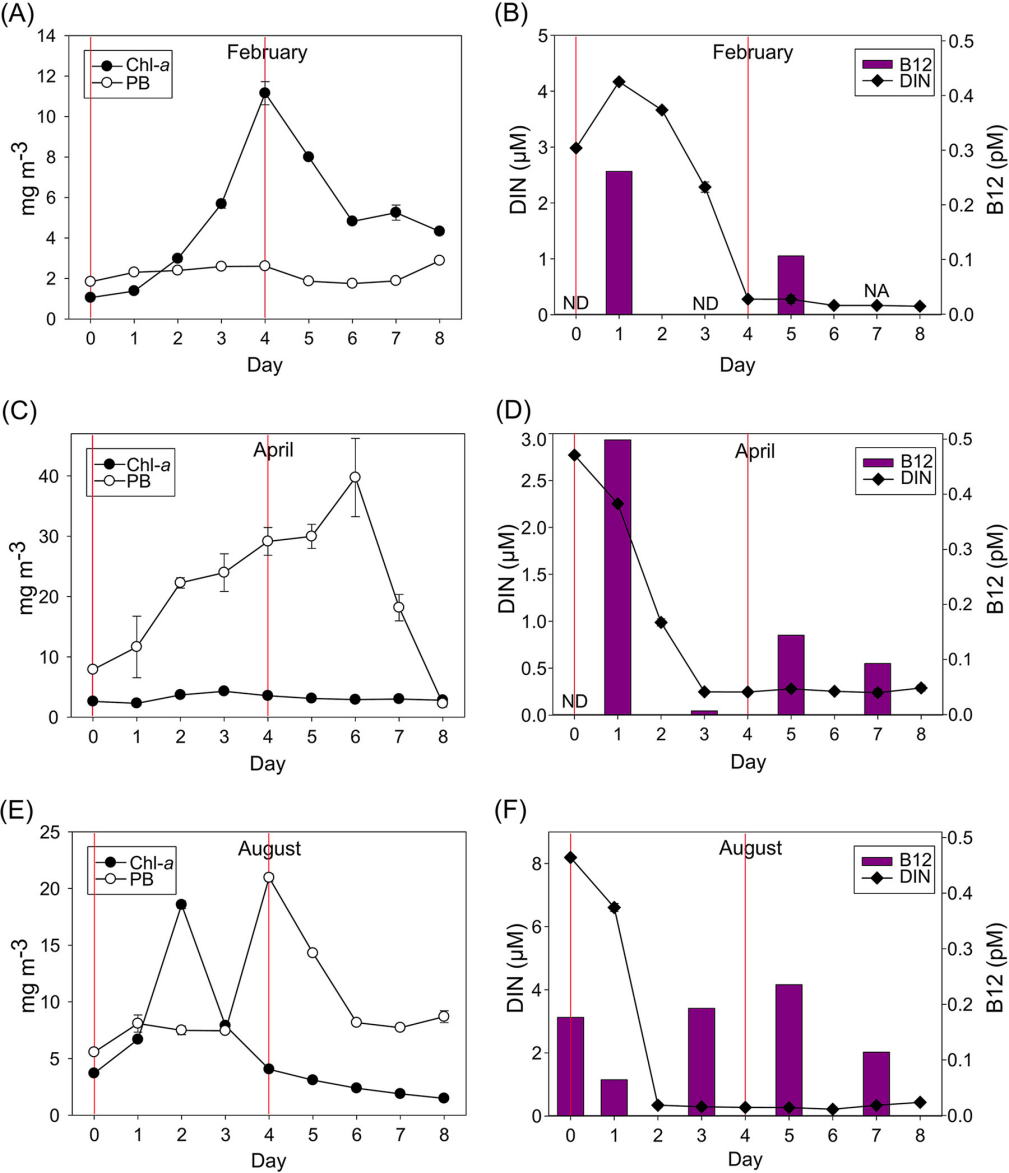
Phytoplankton biomass, estimated as Chl-*a* concentration, prokaryote biomass (PB), dissolved inorganic nitrogen (DIN) and dissolved B_12_ concentration (B_12_) in the mesocosms conducted in February (A and B), April (C and D), and August (E and F). The vertical red lines indicate the beginning of the addition experiments on day 0 (Exp-1) and day 4 (Exp-2). Abbreviations: NA, not available (sample not measured); ND, not detected (sample below detection limit).

There were large differences in microbial dynamics between the three sampling periods (Fig. 1). In February, Chl-*a* increased exponentially from day 1 until day 4, whereas prokaryote biomass (PB) remained rather stable and below 4 mg C m^−3^ throughout the 8-day sampling period (Fig. 1A). The level of dissolved inorganic nitrogen (DIN) was highest on day 1, coinciding with maximum B_12_ concentrations, and decreased to below 0.5 μM on day 4 (Fig. 1B). In contrast, in April, Chl-*a* increased slightly, remaining below 5 mg m^−3^, and prokaryote biomass (PB) increased until day 6, when it reached its maximum (Fig. 1C). B_12_ concentration was relatively high at the beginning of the incubation, dropped to close to undetectable levels on day 3, and slightly increased thereafter, coinciding with a DIN drop below 0.5 μM (Fig. 1D). In August, a peak of Chl-*a* on day 2 was followed by a peak of PB on day 4 (Fig. 1E). At the beginning of the incubation, the highest DIN concentrations were observed, but DIN sharply dropped below 0.5 μM on day 2 (Fig. 1F). B_12_ concentration remained between 0.1 to 0.25 pM during the experimental period (Fig. 1F).

Analysis of microbial community composition using 16S and 18S rRNA sequencing showed different microbial community successions in the mesocosms in the three seasons (see Fig. S2 in the supplemental material). In February, SAR11 and archaea were largely outcompeted by *Synechococcus* and *Alphaproteobacteria*, and a bloom of Ciliophora occurred at the end of the incubation (Fig. S2). In April, the eukaryote community, originally dominated by heterotrophic or mixotrophic taxa (Cercozoa, marine stramenopiles [MAST], and Dictyochophyceae), sharply shifted to a community dominated by autotrophic taxa like *Ostreococcus* and other Chlorophyta (Fig. S2). In the August mesocosms, a bloom of *Tenacibaculum* and *Chaetoceros* occurred (Fig. S2).

### Chlorophyll *a* and prokaryote biomass responses to nutrient additions.

**FIG 2:**
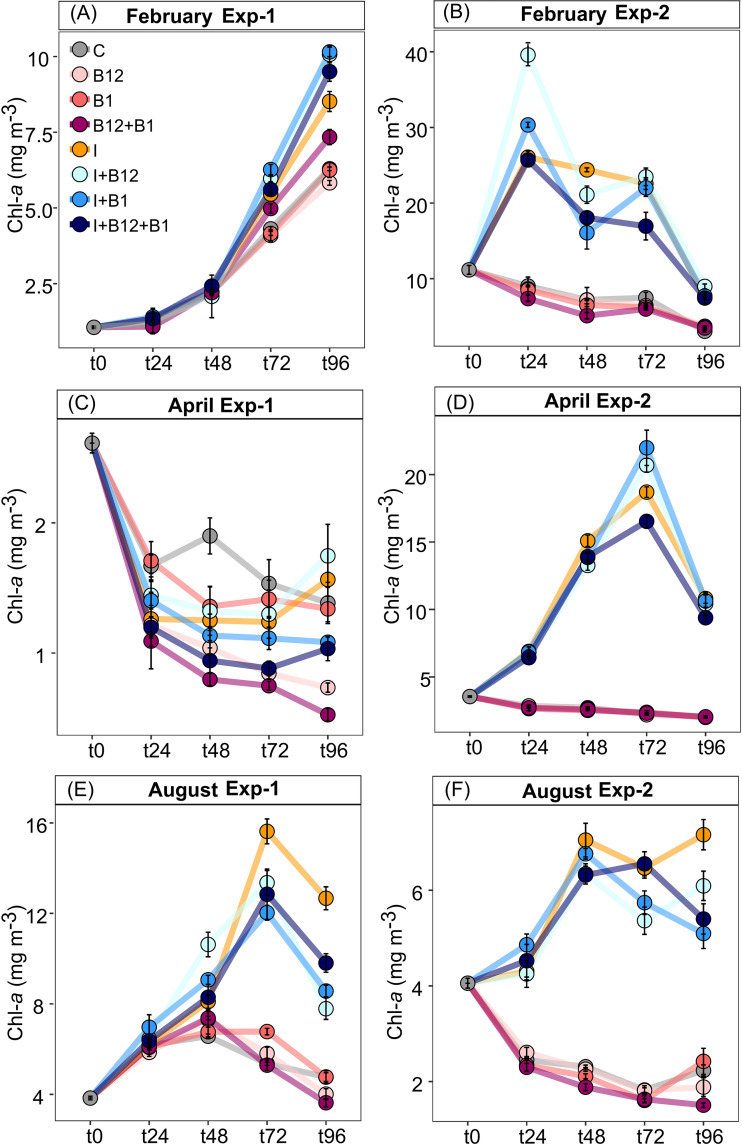
Time course of mean Chl-*a* concentration in samples exposed to different treatments in Exp-1 and Exp-2 in February (A and B), in Exp-1 and Exp-2 in April (C and D), and in Exp-1 and Exp-2 in August (E and F). Error bars represent standard errors.

The Chl-*a* response in experiment 1 (Exp-1) varied between months: important increases were observed in February and August, while a general decrease was registered in April (Fig. 2C). Exp-2, conducted under DIN depletion conditions (Fig. 1B, D, and F), showed Chl-*a* increases in all the inorganic nutrient-containing treatments (Fig. 2B, D, and F).

**FIG 3:**
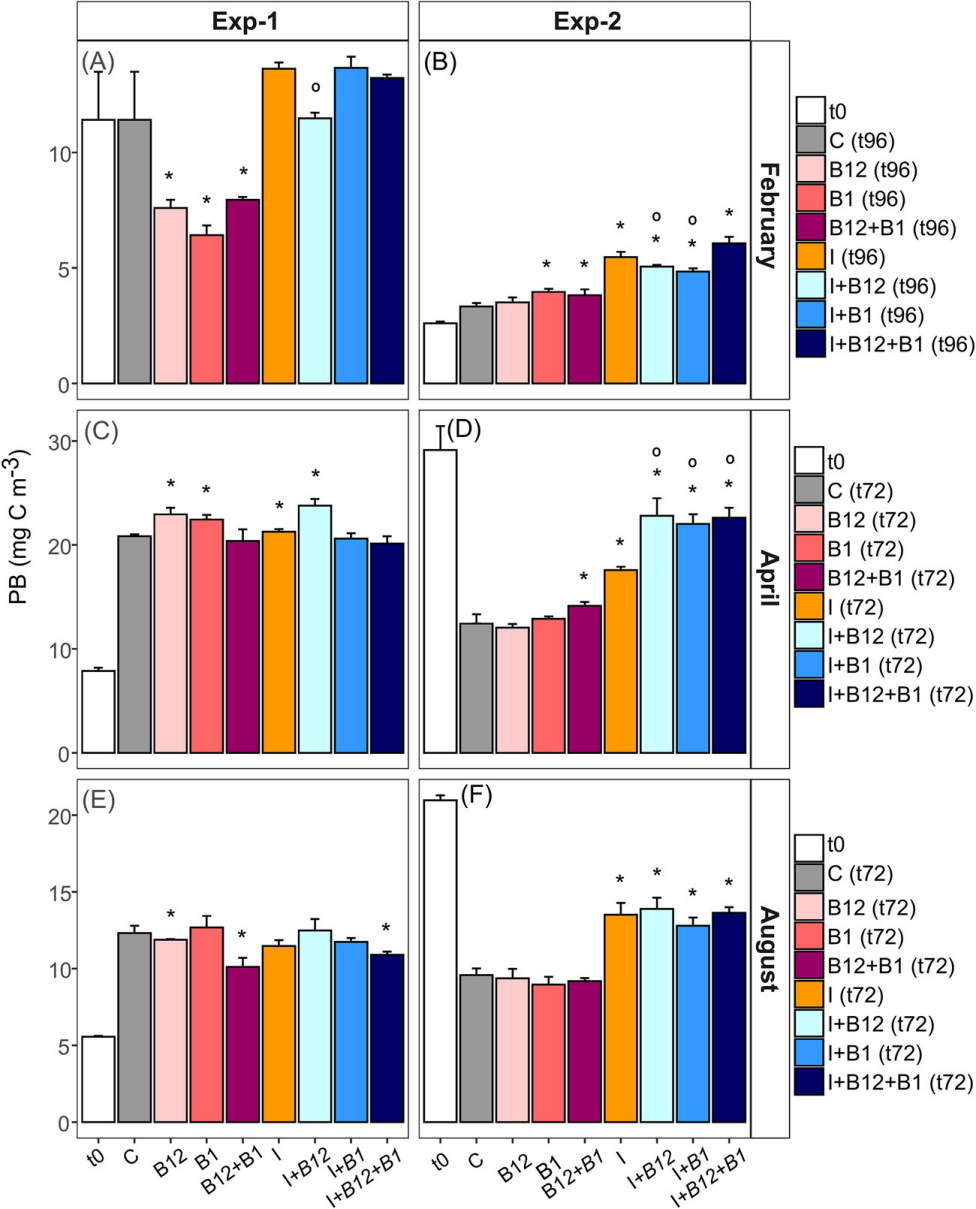
Prokaryote biomass at t0 of each experiment (white bars) and after the incubation of each treatment (colored bars) in Exp-1 and Exp-2 in February (A and B), April (C and D), and August (E and F). Error bars represent standard errors. Asterisks indicate significant prokaryotic primary responses (i.e., between amendments and control) (*t* test; *, *P < *0.05), and circles indicate significant prokaryotic secondary responses (i.e., between amendments and inorganic nutrient treatment) (*t* test; ○, *P < *0.05). Note that different scales were used.

In Exp-1 in February, Chl-*a* increased significantly more than in the controls after combined B vitamin and inorganic amendments (*t* test at t96 [i.e., after 96 h of incubation], *P < *0.05) (Fig. 2A), whereas treatments with either B_1_ or B_12_ alone did not affect phytoplankton growth (Fig. 2A). Also, the addition of B_12_ combined with B_1_ (B_12_+B_1_) triggered a significant increase in Chl-*a* (*t* test at t96, *P = *0.018). In Exp-2, after 24 h of incubation, Chl-*a* increased upon inorganic amendments, while Chl-*a* in the control and B-vitamin treatments progressively decreased over time, suggesting a strong inorganic nutrient limitation (Fig. 2B). At the endpoint of this experiment, significant differences were found in Chl-*a* concentration between all treatments containing inorganic nutrients (I, I+B_12_, I+B_1_, and I+B_12_+B_1_) and the control (*t* test, *P < *0.05). In Exp-1 in April, Chl-*a* concentration decreased over time in all treatments and in the controls, especially in the B_12_+B_1_ treatment (Fig. 2C). In contrast, in Exp-2, at t96, a large increase in Chl-*a* concentration relative to the control was observed in all the treatments containing inorganic nutrients (*t* test, *P < *0.05) (Fig. 2D). Interestingly, the highest Chl-*a* concentrations were observed at t72 when inorganic nutrients were combined with B_1_ or B_12_. Chl-*a* concentrations in the B-vitamin treatments remained similar to those in the control (Fig. 3D). In Exp-1 in August, after t48, the Chl-*a* concentration increase was strongly limited by inorganic nutrients, being significantly higher in the treatments containing inorganic nutrients than in the control at t72 and t96 (*t* test, *P < *0.05) (Fig. 2E). Instead, in Exp-2, the limitation by inorganic nutrients was visible during the entire experiment (*t* test, *P < *0.05) (Fig. 2F). In both experiments conducted in August, at t96, the addition of B_12_ combined with B_1_ had a negative effect on the Chl-*a* concentration compared with the control (*t* test, *P < *0.001).

In February, PB in the control remained stable during the incubation (t96 versus t0) (Fig. 3A and B). In April and August, after 72 h of incubation, PB in the control increased in Exp-1 (Fig. 3C and E) but decreased in Exp-2 (Fig. 3D and F).

In the Exp-1 in February and August, B-vitamin amendments caused significant decreases in PB compared with the control (*t* test, *P < *0.05), while in Exp-1, in April, slight increases were observed in the B_12_ and B_1_ treatments compared with the control (*t* test, *P = *0.018 and *P = *0.029, respectively) (Fig. 3A, C, and E). PB systematically increased after inorganic nutrient addition in Exp-2 in February, April, and August (*t* test, *P < *0.05) (Fig. 3B, D, and F). A significant increase in PB in treatments including both inorganic nutrients and B_12_ and/or B_1_ compared to treatments containing only inorganic nutrients occurred in Exp-2 in April, which was indicative of secondary limitation by B vitamins (Fig. 3D).

### Changes in the microbial community composition.

Changes in prokaryote and eukaryote community composition were analyzed at the endpoints of Exp-1 and Exp-2 in February, of Exp-2 in April, and of Exp-1 in August. We selected those experiments because of the contrasting phytoplankton biomass response patterns (phytoplankton sharply decreased in Exp-1 in April, and Exp-1 and Exp-2 in August presented similar response patterns). The prokaryote diversity was lowest in February and highest in August (Table S3). Specifically, prokaryote diversity varied from 3.73 for the “I” treatment in Exp-2 in February to 4.86 for the I+B_1_ treatment in Exp-1 in August. A wider range was observed for eukaryote diversity, which ranged between 3.20 and 5.08. The lowest eukaryote diversity was observed when B_12_+B_1_ were added in Exp-1 in February. However, the highest diversity was measured in the I+B_1_ treatment of Exp-1 in August.

**FIG 4:**
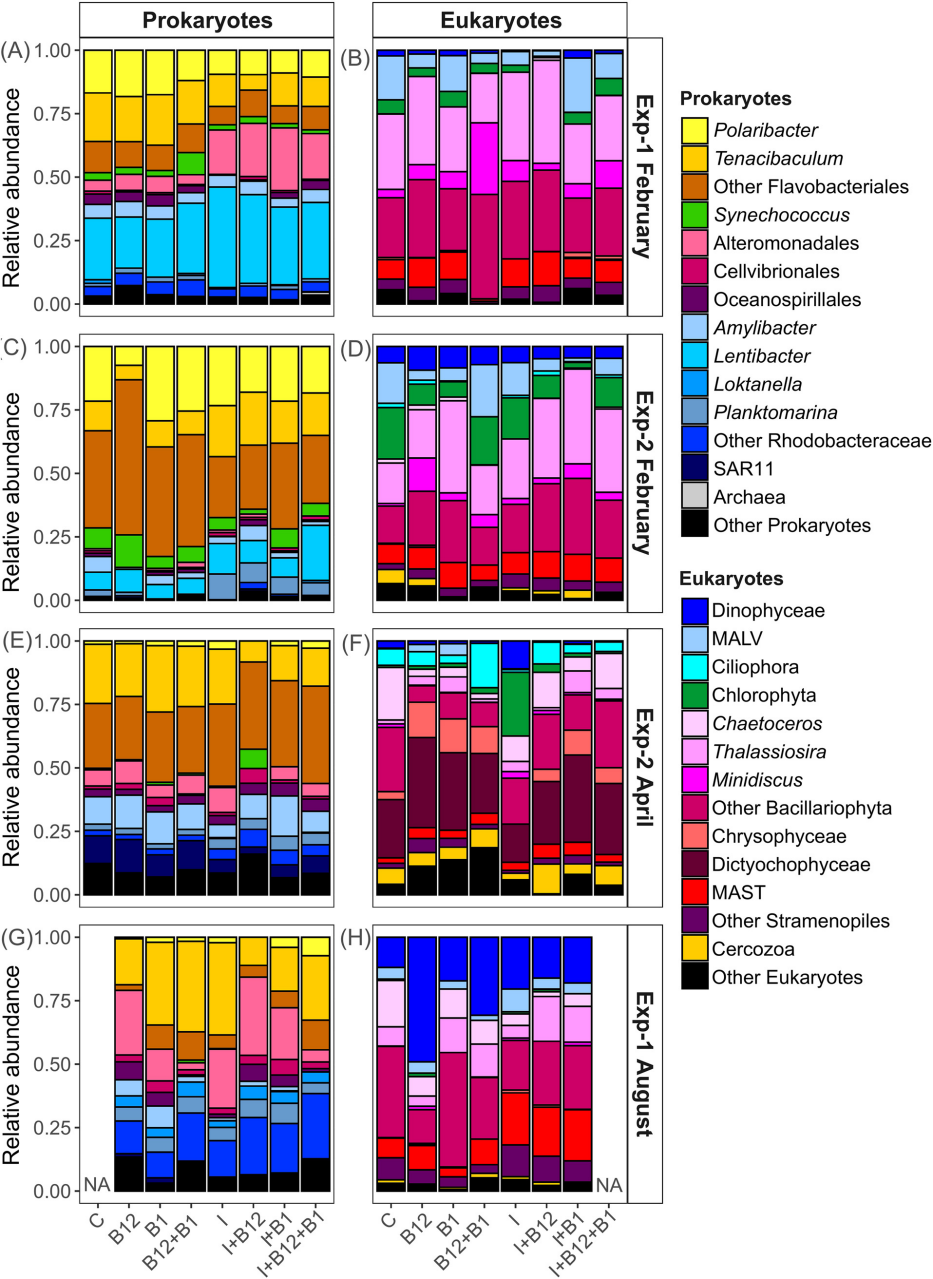
Relative abundance of sequence reads assigned to the major taxonomic groups of prokaryotes and eukaryotes at the endpoint (t96) of Exp-1 (A and B) and Exp-2 (C, D) conducted in February, Exp-2 conducted in April (E and F), and Exp-1 conducted in August (G and H). NA, not available due to failed amplification.

Microbial community composition differed substantially between the t0 (Fig. S2) and the control treatment at t96 (Fig. 4) of each experiment. At the level of major taxonomic groups (key genera and orders), no drastic changes in community composition were observed in response to nutrients and/or B-vitamin amendments (Fig. 4). In Exp-1 in February, *Lentibacter* and *Alteromonadales* increased in relative abundance, whereas *Flavobacteriales*, *Polaribacter*, and *Tenacibaculum* decreased when inorganic nutrients were added (Fig. 4A). In addition, a slight increase in *Synechococcus* relative abundance was observed after B_12_+B_1_ additions. In Exp-2 in February, some interesting changes occurred in the prokaryote community. On one hand, a diverse set of representatives of *Flavobacteriales* other than *Polaribacter* and *Tenacibaculum*, grouped as “other *Flavobacteriales*,” were relatively more present in the B_12_ treatment (Fig. 4C). On the other hand, inorganic nutrients favored an increase in the relative abundance of *Rhodobacterales* such as *Planktomarina* and *Lentibacter* (Fig. 4C). In Exp-2 in April, the composition of prokaryotes did not show clear changes upon addition of nutrient supplements (Fig. 4E). Remarkably, *Synechococcus* was mostly present in the I+B_12_ treatment. In the case of eukaryotes, the most pronounced changes were the increase in the relative abundance of Ciliophora in the B_12_+B_1_ treatment, the increase in the relative abundance of Chrysophyceae together with the decrease in the relative abundance of *Chaetoceros* in the B vitamin treatments, and the increase in the relative abundance of Chlorophyta in the inorganic-nutrient treatment (Fig. 4F). In Exp-1 in August, the relative abundance of *Flavobacteriales* was reduced in treatments containing only B_12_ compared to other enrichment treatments (Fig. 4G). Unfortunately, data on the control prokaryote community are not available for this experiment (Fig. 4G). Concerning the eukaryote community, diatoms (Bacillariophyta) and Dinophyceae dominated in all treatments (Fig. 4H). Interestingly, Dinophyceae dominated the eukaryote community in the B_12_ treatment (Fig. 4H).

**FIG 5:**
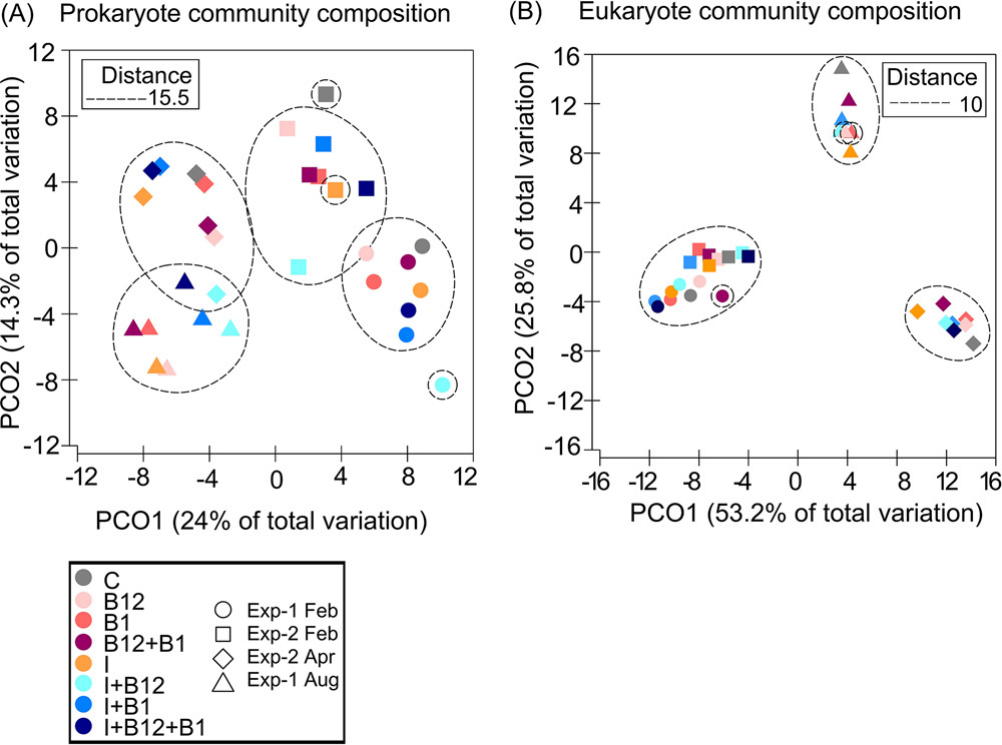
Principal-coordinate analysis (PCoA) of the Euclidean distance matrix of the (A) prokaryote and (B) eukaryote community composition at the endpoint of each treatment in Exp-1 (circles) and Exp-2 (squares) in February, in Exp-2 in April (diamonds), and Exp-1 in August (triangles). The two first axes represent 38.3% and 79% of the total variation in the prokaryote and eukaryote community composition, respectively. Dotted circles represent the minimum Euclidean distance between samples included in them. The represented distance is 15.5 for prokaryotes and 10 for eukaryotes.

Considering the prokaryote and eukaryote community data at the amplicon sequence variant (ASV) level, principal-coordinate analysis (PCoA) based on Euclidean distances at the endpoint of each experiment revealed that samples clustered by experiments (Fig. 5). Accordingly, significant differences in both prokaryote and eukaryote communities were observed between experiments (analysis of similarity [ANOSIM], *P < *0.001) (Fig. 5). The eukaryotic community composition varied between months, suggesting a strong effect of the initial microbial community. In the case of prokaryotes, samples were more overlapped between months, suggesting a certain response to treatments. Overall, the mean Euclidean distance among samples from the same experiment was larger for prokaryotes than for eukaryotes (Fig. 5).

The first principal coordinate explained 24.0% and 53.2% of total variation in the prokaryote and eukaryote communities, respectively, and the second principal coordinate explained 14.3% and 25.8% of total variation in prokaryote and eukaryote communities, respectively. The two main coordinates explained a larger fraction of the variance of eukaryotes (79%) than prokaryotes (about 38%), probably because the composition of eukaryotes was totally different in the 3 samplings, and this was not the case for the prokaryotes. In February, the prokaryote community differed clearly between the two consecutive experiments (Fig. 5A), and the eukaryote community from these two experiments was more similar yet slightly shifted (Fig. 5B). Curiously, in both Exp-1 and Exp-2 in February and in Exp-2 in April, the largest shifts in prokaryote composition occurred in the I+B_12_ treatment (Fig. 5A).

**FIG 6:**
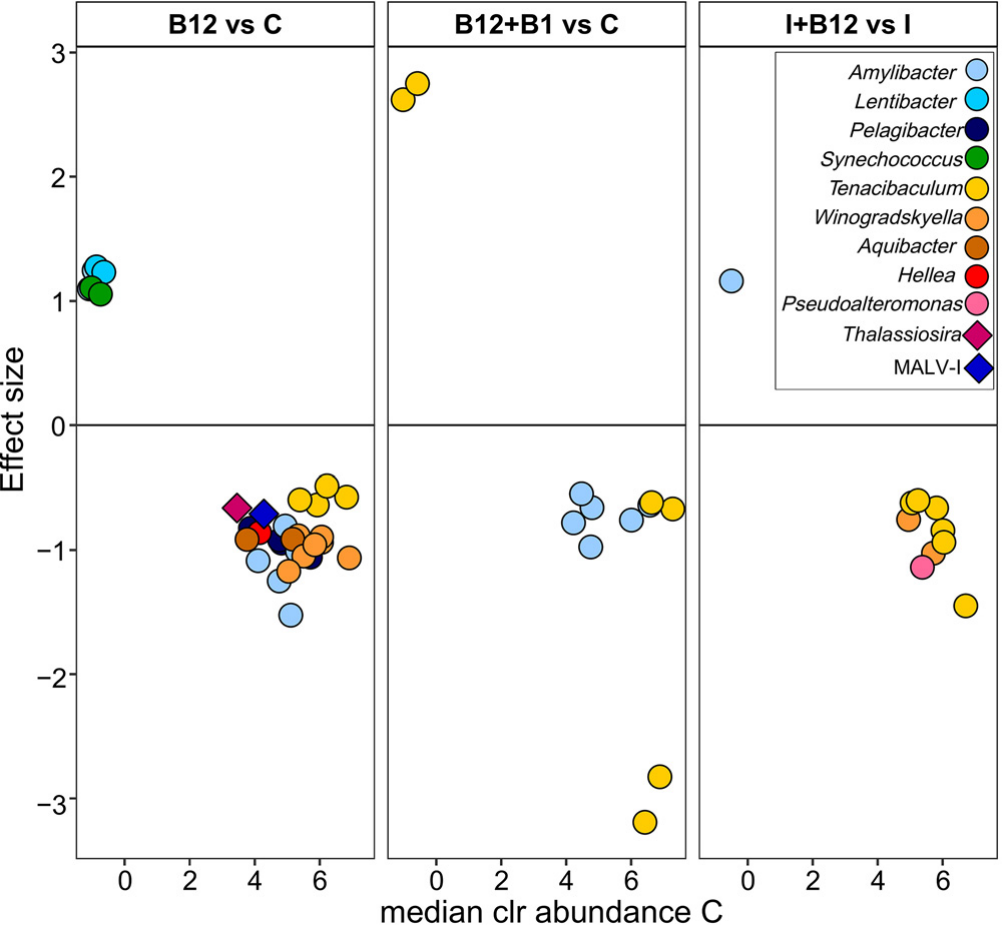
Prokaryote (circles) and eukaryote (diamonds) ASVs that significantly varied in abundance after the addition of B vitamins compared to control (C) or inorganic treatment (I). The graph shows the clr abundance in the control or inorganic treatment (*x* axis) and the effect of vitamins (*y* axis). An effect size of <0 indicates that the ASV abundance significantly decreased in the vitamin treatments, and an effect size of >0 indicates that the ASV abundance increased in the vitamin treatments. The *x* axis represents the median clr value for each ASV in the control treatments, and *y* axis values were calculated as the median of the ratio of the between treatment difference and the larger of the variance within treatments.

The ALDEx test was performed to identify populations, as defined by ASVs, which significantly changed in relative abundance to B vitamin additions (Fig. 6). Several ASVs taxonomically classified as *Alphaproteobacteria*, *Cyanobacteria*, *Flavobacteriaceae*, and *Gammaproteobacteria* (ALDEx, *P < *0.05; false discovery rate [FDR] < 0.05) showed significantly different relative abundances between B_12_ or B_12_+B_1_ and the control treatments and between the I+B_12_ and I treatments (Fig. 6). In contrast, only two eukaryote ASVs showed significant differences between B_12_ and the control (ALDEx, *P < *0.05; FDR < 0.05) (Fig. 6). The magnitude and sign of the response seemed to be associated with their relative abundance in the control rather than with the taxonomic affiliation. The addition of vitamins favored ASVs with low abundance in the control, whereas the effect was the opposite for ASVs with higher abundance in the control. Only a few ASVs assigned to *Lentibacter*, *Amylibacter*, *Synechococcus*, and *Tenacibaculum* were positively affected (i.e., increased in abundance) after B_12_ or B_12_+B_1_ addition (Fig. 6). The abundance of eukaryote ASVs assigned to *Thalassiosira* and MALV-I was reduced after B_12_ addition.

### Changes in the bacterial gene expression.

We selected Exp-1 in February to perform the gene expression analysis as it was the only experiment showing sustained phytoplankton growth in all treatments. Nonmetric multidimensional scaling (NMDS) analysis of all genes showed that the inorganic nutrient treatment was the most distant compared to the control (Fig. S3). Of the 39 overall metabolic functions (SEED categories) into which genes were grouped (Table S1), 9 SEED categories showed interesting differences in the relative abundance of reads (in counts per million [cpm]) between treatments and controls (Fig. S4). The SEED categories “motility and chemotaxis,” “photosynthesis,” “stationary phase,” “plastidial electron transport,” and “plant cell walls” showed higher relative abundances in the vitamin B_12_ and B_1_ treatments. The single addition of B vitamins (i.e., B_12_ or B_1_) resulted in slightly higher relative abundance in the SEED categories “cell signaling,” “phages and plasmids,” “central metabolism,” and “secondary metabolism” (Fig. S4). Overall, such responses were attenuated when B vitamins were added in combination with inorganic nutrients (Fig. S4). Genes involved in “central metabolism,” “secondary metabolism,” and “plant cell walls” were relatively more abundant in the “I” treatment.

**FIG 7:**
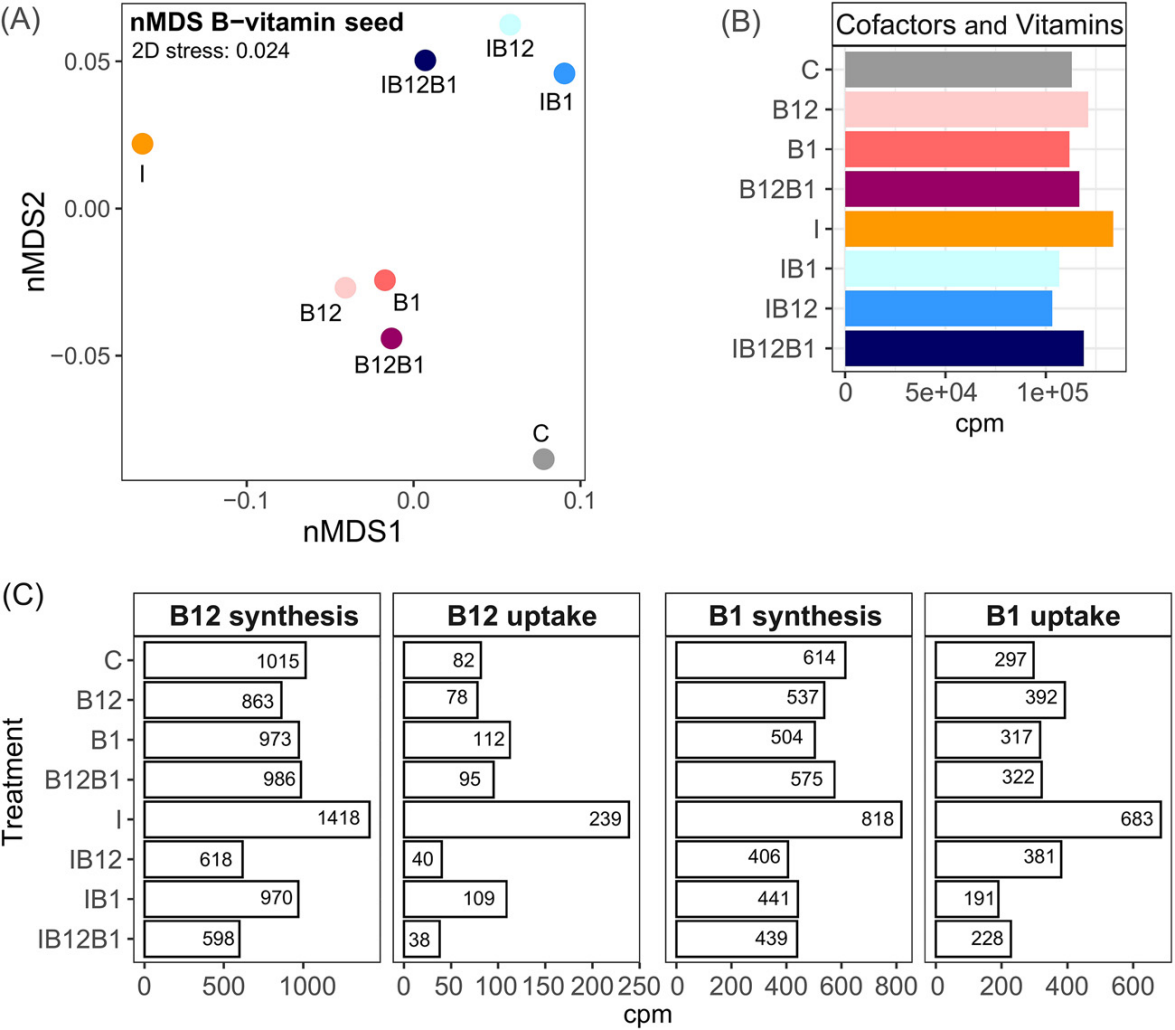
(A) NMDS showing the Euclidean distance according to similarity between treatments in the bacterial gene expression involved in B-vitamin metabolism at the end of Exp-1 in February. (B) Relative abundance of genes within the SEED category “cofactors and vitamins” for each treatment at the end of Exp-1 in February. (C) Proportion of vitamin B_12_ and B_1_ metabolism genes expressed by the bacterial community at the endpoint of each treatment in Exp-1 in February. Counts per million of genes for B_12_ synthesis, B_12_ uptake, B_1_ synthesis, and B_1_ uptake are shown (note different scales on the *x* axis). Colors correspond to the treatments. Detailed lists of genes involved in B_12_ synthesis, B_12_ uptake, B_1_ synthesis, and B_1_ uptake are provided in Tables S2 and S3.

An NMDS analysis of expressed vitamin metabolism genes revealed three distinct groupings, including the group of samples amended with B vitamins, a second one amended with both B vitamins and inorganic nutrients, and the sample where only inorganic nutrients were added (Fig. 7A). More than 100,000 cpm involved in the metabolism of “cofactors and vitamins” were identified in each treatment, showing only slight differences between them (Fig. 7B). Overall, the contribution of B_12_ and B_1_ synthesis genes to the total expressed genes was ca 3.6-fold greater than that of B_12_ and B_1_ uptake genes (Fig. 7C). While the addition of vitamin B_12_ stimulated the expression of genes involved in the uptake of B_1_, the addition of inorganic nutrients resulted in consistently higher relative expression levels of genes related to the synthesis and uptake of B_12_ and B_1_ (Fig. 7C). However, addition of B vitamins together with inorganic nutrients resulted in lower relative expression of genes related to the synthesis and uptake of B_12_ and B_1_ compared to values in the I treatment (Fig. 7C). Notably, treatments with both B_12_ and inorganic nutrients (i.e., I+B_12_ and I+B_12_+B_1_) greatly reduced (∼2-fold) the relative abundance of B_12_ synthesis genes compared to I alone (Fig. 7C). Also, the proportion of genes related to uptake of B_12_ was ∼6-fold lower in I+B_12_ and I+B_12_+B_1_ than in I treatments (Fig. 7C).

**FIG 8:**
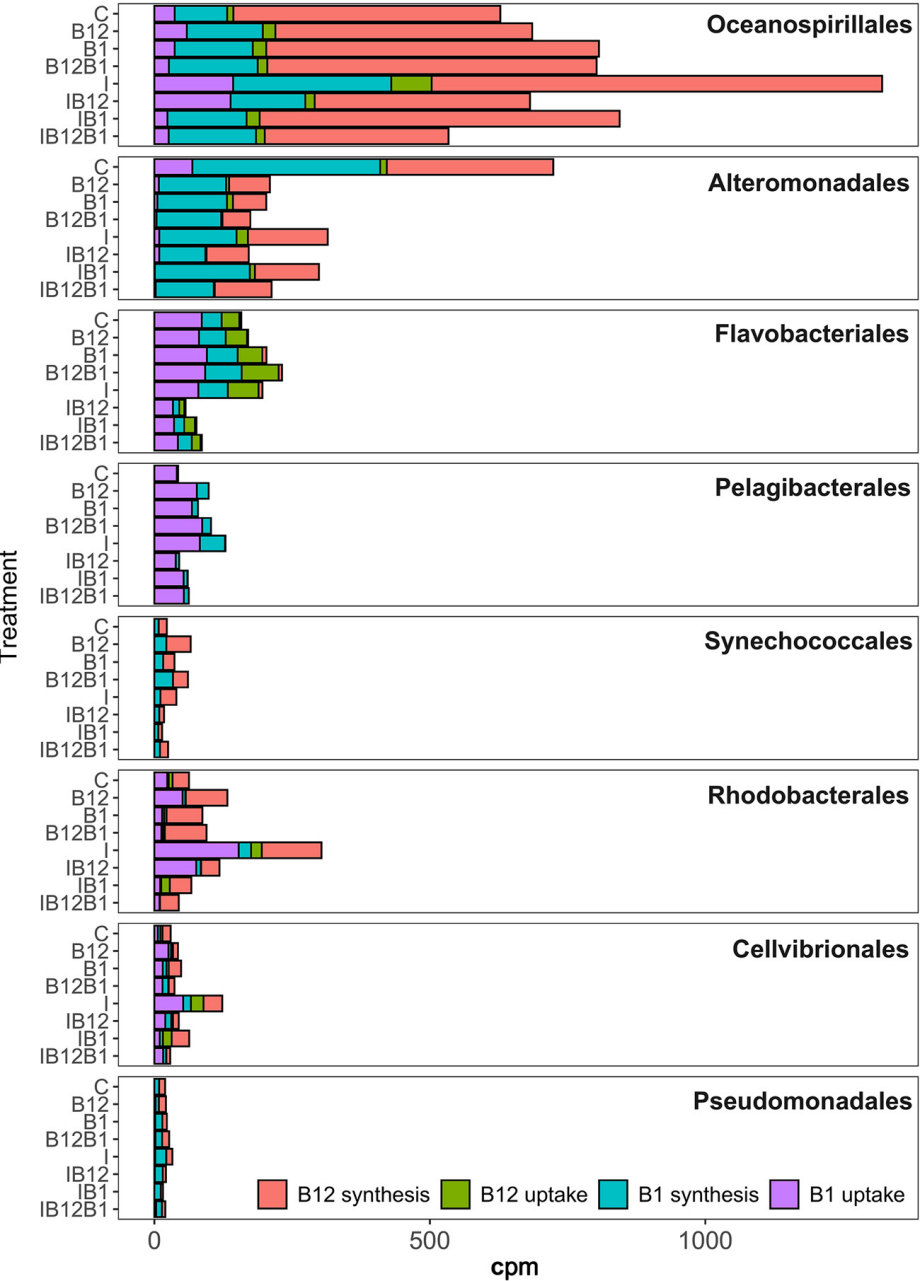
Expression of vitamin B_12_ and B_1_ metabolism genes by different bacterial taxonomic orders in each treatment at the end of Exp-1 in February.

**FIG 9:**
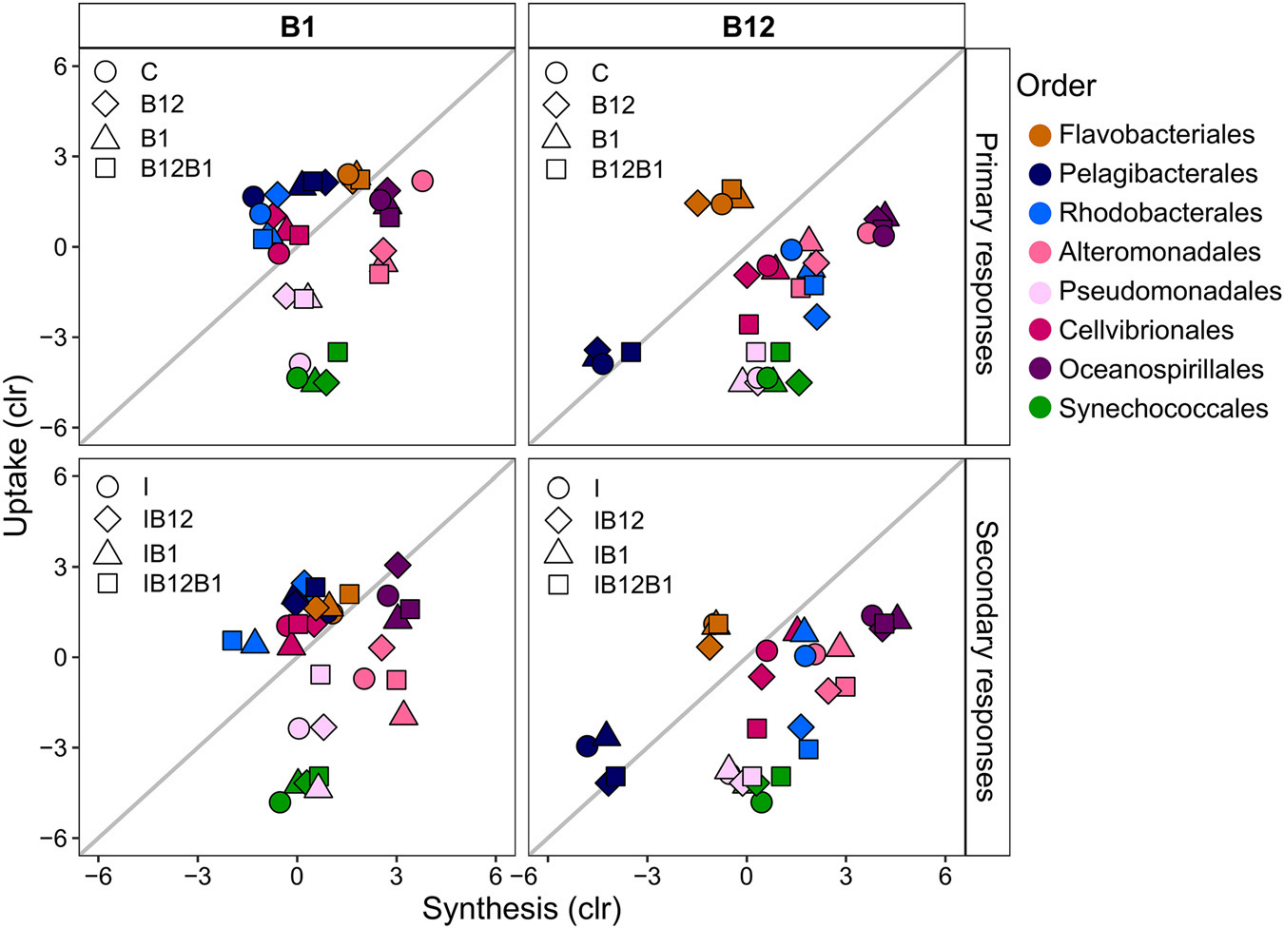
Scatterplot showing the contribution to B_1_ and B_12_ synthesis and uptake of the eight bacterial orders that contributed most to B-vitamin metabolism gene expression at the end of Exp-1 in February. Upper plots represent primary responses to B vitamins and include control (C) treatment, B_12_, B_1_, and B_12_+B_1_. Bottom plots represent secondary responses to B vitamins and include inorganic (I) treatment, I+B_12_, I+B_1_, and I+B_12_+B_1_. Colors correspond to different bacterial taxonomic orders.

The expression of genes related to vitamin B_12_ and B_1_ metabolism differed among members of the bacterial community (Fig. 8). The eight orders that contributed most to the B vitamin metabolism expressed 82% and 87% of the reads of genes for synthesis and uptake of B_1_, respectively, and 85% and 79% of the reads related to synthesis and uptake of B_12_, respectively (Fig. 7C and 8). These orders were the *Oceanospirillales*, *Alteromonadales*, *Cellvibrionales*, and *Pseudomonadales* in the class *Gammaproteobacteria*, *Pelagibacterales* and *Rhodobacterales* in the class *Alphaproteobacteria*, the *Flavobacteriales*, and the *Synechococcales* in the phylum *Cyanobacteria* (Fig. 8). There were differences between the relative contributions of these eight orders in the synthesis and uptake of B_1_ and B_12_ and in their primary (i.e., B-vitamin versus control treatments) and secondary (i.e., I+B-vitamin versus I treatments) responses to B vitamins (Fig. 9).

*Oceanospirillales* contributed most to the relative expression of vitamin genes (Fig. 8). B_12_ and B_1_ appeared to be mostly synthesized by *Oceanospirillales* and *Alteromonadales*. Overall, *Bacteria* taxa (except *Flavobacteriales* and *Pelagibacterales*) contributed more to synthesis than to uptake of B_12_ (Fig. 8 and 9). Only half of the orders (*Flavobacteriales*, *Pelagibacterales*, *Rhodobacterales*, and *Cellvibrionales*) contributed more to synthesis than to uptake of B_1_ (Fig. 8 and 9). While *Flavobacteriales* and *Pelagibacterales* expressed only a few B_12_ synthesis genes, both orders showed higher relative expression levels of genes for B_1_ uptake (Fig. 8 and 9).

Overall, the expression of B_12_ or B_1_ synthesis genes did not change in response to B-vitamin amendments without inorganic nutrients (i.e., primary responses), except in the case of *Pelagibacterales*, where B_1_ synthesis gene expression increased, *Alteromonadales*, where B_1_ uptake and B_12_ synthesis decreased, and *Pseudomonadales*, where B_1_ uptake increased (Fig. 9). Similarly, the addition of B vitamins and inorganic nutrients did not generally alter B_12_ or B_1_ expression profiles compared to the inorganic treatment (i.e., secondary responses) (Fig. 9), except in the case of *Alteromonadales* and *Pseudomonadales*, which tended to increase the expression of genes related to B_1_ synthesis (Fig. 9).

*Rhodobacterales* moderately contributed to the expression of B_12_ synthesis genes (Fig. 8), and the relative abundance of B_12_ uptake genes decreased after B_12_ addition (alone or combined with inorganic nutrients) (Fig. 9).

## DISCUSSION

Surface water from a shelf station off northwestern Spain was enclosed in mesocosms in winter, spring, and summer in order to induce succession in the microbial community over a period of 8 days—i.e., within the time frame of upwelling phytoplankton bloom dynamics (35). The enrichment experiments conducted with mesocosm water allowed the assessment of the microbial responses to vitamin B_12_ and/or B_1_ additions during contrasting phases of microbial community development. The overall low concentration of B_12_, and presumably also of B_1_ (25, 27), contrasts with the overall relatively limited response of phytoplankton and prokaryotes to B_12_ and/or B_1_ amendments, not only in terms of biomass (25) but also in terms of taxonomic composition (this study). On the other hand, while B_12_- and B_1_-related gene expression was not strongly affected by single B_12_ and/or B_1_ additions, important changes were observed upon inorganic nutrient enrichment or when these B vitamins were added in combination with inorganic nutrients. These results support the conception of a microbial community well adapted to the rapid turnover of these compounds in this productive ecosystem.

### Changes in the microbial community composition associated with changes in the microbial biomass.

Enhanced phytoplankton growth associated with B_12_+B_1_ additions in the first experiment in February is consistent with the idea that higher availability of these vitamins may favor phytoplankton growth when inorganic nutrient concentration is high (22, 23, 36). The increase in Chl-*a* in the B_12_+B_1_ treatment was associated with the proliferation of the diatom genus *Minidiscus*. Although B_12_ auxotrophy is widespread in diatoms (4), to our knowledge, the B-vitamin auxotrophy within this genus has not been described so far. On the other hand, genes required for B_1_ biosynthesis have been detected in diatoms, suggesting that a majority of diatoms produce B_1_ (5, 17, 37). The consistent decrease in prokaryote biomass associated with single B_12_ and/or B_1_ additions during Exp-1 in February could be related to predation or competitive interactions with larger auxotrophic phytoplankton. MAST representatives, which have been described as bacterivores (38), were abundant during this experiment, and their activity could be favored by the B-vitamin enrichment. It is well known that when inorganic nutrients are abundant, the larger phytoplankton, such as diatoms, have a competitive advantage over smaller species, for example, prokaryotes. However, prokaryote biomass remained stable when inorganic nutrients were added, coinciding with a pronounced increase in the relative abundance of *Alteromonadales*, which are known to rapidly respond to nutrient enrichments (39, 40).

In the second experiment in February, modest increases in prokaryote biomass in response to B_12_ and/or B_1_ amendments seemed to be related to slight increments in the relative abundance of *Flavobacteriales*, a potentially B_12_- and B_1_-auxotrophic group (5, 19).

Strong predation pressure during the incubations may explain the lack of response of phytoplankton biomass observed in Exp-1 in April. This is supported by the observation that the eukaryote community composition at the beginning of this experiment was dominated by Dictyochophyceae, Chrysophyceae, and MAST (Fig. S2), which include heterotrophic or mixotrophic species (38, 41). In the second experiment in April, secondary responses to vitamins B_12_ and B_1_ of both phytoplankton and prokaryote biomass were accompanied by changes in the community composition. In the case of phytoplankton, the higher relative abundance of the class Chrysophyceae (phylum Ochrophyta) in the treatments containing B vitamins strongly suggests that auxotrophy may be widespread within this taxon. This is consistent with the observations of in reference 7, which reported that all or three-fourths of the studied Ochrophyta species required exogenous B_12_ or B_1_, respectively. Interestingly, heterotrophic eukaryotic taxa, such as Ciliophora and Cercozoa, were also more abundant in the treatments containing both inorganic nutrients and B vitamins than in the one containing only inorganic nutrients, which could be related to the higher biomass of phytoplankton accumulated in the combined treatments up to t72.

*Synechococcus*, which is considered a major producer of B_12_ (5, 31), considerably increased in relative abundance in the I+B_12_ treatment in Exp-2 in April, although the reason for this response is not clear. Specifically, these bacteria synthesize only pseudocobalamin, in which the lower axial ligand is adenine, instead of 5,6-dimethylbenzimidazole (DMB) cobalamin used by algae (31). We raise the possibility that *Synechococcus* may remodel B_12_ to synthesize pseudocobalamin.

In Exp-1 in August, the significant decreases in prokaryote biomass in treatments containing B_12_ could be associated with the relatively higher abundance of Dinophyceae. Many Dinophyceae species are auxotrophs for B_1_ and/or B_12_ (4, 7), and many of them are also mixotrophs and, therefore, may be predators of marine bacteria (42–44). The B_12_-dependent enzyme methylmalonyl coenzyme A (methylmalonyl-CoA) mutase in mixotrophs allows them to grow heterotrophically when B_12_ is available (4). Thus, high bioavailability of B vitamins might promote the growth of heterotrophic and/or mixotrophic species, causing a decrease in prokaryote biomass at the end of the experiments. This is consistent with the results obtained in three short-term microcosm experiments conducted with field samples during the summer cruise (25).

Overall, the eukaryote community composition was relatively less affected by the additions of B vitamins and inorganic nutrients than the prokaryote community composition in this productive region. This suggest that eukaryotes may obtain B vitamins through biotic relationships being more dependent on the existence of close interactions among other microorganisms (such as mutualism or predation) than on exogenous inputs (7, 45–48). Among the heterotrophic bacteria, the addition of vitamin B_12_ mostly had a negative effect on *Rhodobacterales* (presumably prototrophic [32]) and *Flavobacteriales* (presumably auxotrophic [5]) populations. The fact that the negative impact of B vitamins was particularly pronounced among relatively abundant taxa points to an indirect effect, implying a stimulation of bacterivores that in turn would forage on the most abundant groups. The decrease in *Rhodobacterales* when B-vitamin concentrations are high could subsequently affect autotrophic algae by altering mutualistic interactions whereby bacteria supply B_12_ to the algae in exchange for fixed carbon (46, 49).

Many prokaryotes can satisfy their biological B_1_ demands only through the uptake of B_1_ precursors (18), for example by recycling the decomposition products of B_1_ (50). On the other hand, larger plankton organisms may obtain this compound through bacterial or phytoplankton predation (51). These alternative processes for obtaining exogenous B_1_, and perhaps others not contemplated here, may explain the lack of significant responses to the addition of B_1_ in microbial populations.

### Changes in the bacterial gene expression after B-vitamin supply.

We acknowledge that the lack of replication precluded statistical analysis of the responses of prokaryotic gene expression to the external supply of B vitamins and nutrients. Nevertheless, the qualitative assessment of results across sets of treatments (e.g., vitamins with or without inorganic nutrients) provided valuable insight into the functional responses of prokaryote populations. Although the addition of B_12_ or B_1_ without inorganic nutrients caused a significant decrease in prokaryote biomass and minor changes in prokaryote community composition (Exp-1; February), many metabolic categories showed higher relative abundances in these treatments. This suggests an uncoupling between biomass, diversity, and functional responses upon environmental changes and implies a need to conduct integrative studies to properly assess the role of abiotic factors in microbial dynamics. Genes encoding cobalamin (52, 53) or thiamine (54) transporters can also encode transporters of B vitamin precursors (55–58) to salvage these vitamins (for example, see references 59 and 60). Expression of genes for B_12_ synthesis and uptake had a tendency to reach lower relative abundances when B_12_ in combination with inorganic nutrients were added, while relative expression levels were higher when only inorganic nutrients were added. These results suggest an increased B_12_ demand associated with high microbial biomass in the inorganic nutrient treatment (I), which would cause an increase in the expression of genes involved in B_12_ synthesis and uptake. Such high B_12_ demand could be reduced when B_12_ is externally supplied. It has been observed that cobalamin transporters decrease under B_12_ replete conditions (61). The increase in the expression of B_12_ and B_1_ synthesis genes associated with inorganic nutrient addition points to a link between inorganic nutrient availability and vitamin supply. This suggests that the microbiome associated with phytoplankton could maintain an adequate vitamin supply to exploit the inorganic nutrients intermittently reaching the photic zone in this upwelling ecosystem.

The observation that *Oceanospirillales* and *Alteromonadales* were responsible for more than 70% of the expression of genes for synthesis of vitamins in the unamended control at the endpoint of Exp-1 in February indicated that these taxa might be potential producers of B_1_ and B_12_, respectively. This is consistent with previous observations in marine surface waters, where *Oceanospirillales* appeared to be major B_12_ synthesizers (32, 62). However, to the best of our knowledge, *Alteromonadales* have never been considered an important source of vitamins, although their potential to synthesize B_1_ has been suggested from genomic data (5). Prokaryote biomass was reduced when B vitamins were added, even though the bacterial gene expression associated with B_12_ remained fairly stable, suggesting the maintenance of B_12_ metabolism in prokaryotes after B-vitamin addition. In addition, the high contribution to B_12_ synthesis gene expression by *Oceanospirillales* generally appeared stable in all treatments, which brings to light this rare group (representing on average <3% of the total 16S rRNA sequences) as a potential main producer of B_12_ in this region during winter, regardless of B-vitamin or nutrient availability. Despite the consistent increase in the relative abundance of 16S rRNA sequences belonging to *Alteromonadales* in all the treatments containing inorganic nutrients, the contribution to synthesis and uptake of B_12_ and B_1_ did not increase, suggesting that B-vitamin metabolism in this group was not stimulated by the addition of inorganic nutrients.

*Rhodobacterales*, one of the best-represented taxa during the experiment, were expected to greatly contribute to B_12_ synthesis (19, 32, 62) and, to a lesser extent, to B_1_ synthesis (5). However, in contrast to the model systems where *Rhodobacterales* support the growth of phytoplankton in B_12_-deficient media (63), the relative abundance of vitamin B_12_ synthesis genes associated with *Rhodobacterales* was relatively low in waters off northwestern Spain. Vitamin supply slightly increased the contribution of *Rhodobacterales* to B_12_ synthesis, suggesting that this group could incorporate B_12_ precursors that enter the salvage synthesis route, as previously suggested (60, 64).

*Flavobacteriales* and *Pelagibacterales* contributed more to B_12_ and/or B_1_ uptake than to synthesis. In the present work, *Flavobacteriales*, which are expected to be B_12_ auxotrophs, were potentially the main consumers of B_12_, which is consistent with their predicted inability to conduct *de novo* B_12_ synthesis (5, 60) and their strong dependence on external B_12_ supply (5, 19). The relative contribution of *Flavobacteriales* to B_12_ synthesis was extremely low, which is consistent with a recent review reporting that only 0.6% of this group produces B_12_ *de novo* (60).

*Pelagibacterales* genomes have incomplete pathways for *de novo* B_1_ synthesis, so they need to incorporate B_1_ precursors (18). The relative contribution of this group to B_1_ synthesis and uptake gene expression tended to increase when vitamins were added. However, their relative contribution showed a tendency to decrease when vitamins and inorganic nutrients were added compared with the addition of inorganic nutrients alone. This is surprising, as SAR11 is supposed to neither require B_12_ nor have pathways for its synthesis (65); thus, the connection between B_12_ external supply and B_1_ metabolism remains unclear.

As mentioned above, *Synechococcales* produce the B_12_ analog pseudocobalamin (31). Accordingly, genes for B_12_ *de novo* biosynthesis have been found in the majority of *Synechococcales*, except genes coding for lower axial ligand (5, 31). Also, all *Cyanobacteria* seem to be able to produce B_1_ (37). In the present work, *Cyanobacteria* contributed marginally to the expression of B_12_ or B_1_ uptake genes, which points to a generalized ability among *Cyanobacteria* to produce both B vitamins *de novo*.

### Conclusions.

Overall, our results confirm that initial abiotic conditions and initial microbial community composition seem to be major factors determining the microbial responses associated with B-vitamin amendments (17, 23). Importantly, changes in Chl-*a* or prokaryote biomass in response to enrichments were not always accompanied by changes in taxonomic composition or in the expression of B-vitamin-related genes. This implies that the response of microbial plankton to vitamin availability should be addressed from different perspectives considering the different field nutritional conditions (inorganic nutrients, metals, and other organic compounds not contemplated here) and/or the abundance of heterotrophic protists and metazoan zooplankton to fully understand the complex community dynamics. The diverse responses in B-vitamin metabolism within the bacterioplankton observed in this investigation suggest that the availability of these growth factors and their precursors might contribute to niche differentiation, likely playing a significant role in determining the structure and function of marine microbial communities.

## MATERIALS AND METHODS

### Survey area.

The Ría de Vigo (northwestern Spain) is a coastal embayment affected by intermittent upwelling of cold and inorganic nutrient-rich subsurface water from April to September and downwelling of warm and nutrient-poor shelf surface water from October to March. The Ría de Vigo and its adjacent shelf constitute a highly productive and exceptionally dynamic coastal system, where microbial community composition varies over short temporal and spatial scales (66).

### Experimental procedures.

Data and samples included in this study were collected on board the B/O Ramón Margalef during three oceanographic cruises within the ENVISION project conducted in 2016. The first cruise was carried out from 17 to 26 February, the second cruise was carried out from 16 to 25 April, and the last cruise was carried out from 5 to 14 August.

During each cruise, a microbial succession experiment was performed in on-board mesocosms with surface water from a coastal station (42.14° N, 8.88° W). For this experiment, 190 liters (in triplicate) of seawater was incubated at *in situ* light and temperature for 8 days using 208-liter low-density polyethylene cylindrical tanks, which were placed on deck in a 4.1-m^3^ rectangular tank (2.3 by 1 by 1.8 m) where surface seawater was continuously circulating. The enclosed seawater was collected at a 5-m depth in February and April, while 152 liters of 20-m-depth seawater was mixed with 38 liters of 5-m-depth seawater in August, in order to simulate an upwelling episode. Seawater samples from each replicate mesocosm were taken daily for dissolved inorganic nitrogen (DIN), prokaryote biomass (PB), and chlorophyll *a* (Chl-*a*) analyses. Dissolved B_12_ concentration was measured in each mesocosm on days 0, 1, 3, 5, and 7. Additionally, 2 liters from each replicate mesocosm were taken for microbial plankton community composition analyses by partially sequencing 16S and 18S rRNA gene on days 0, 1, 3, 5, and 7.

To evaluate microbial community composition and prokaryotic functional responses to B-vitamin and nutrient amendments, addition experiments with homogeneous mixtures of the three replicate mesocosms were conducted during each cruise. Based on expected changes in nutrient concentrations during mesocosm incubations (primarily the drawdown of inorganic nitrogen), addition experiments with vitamins and inorganic nutrients were conducted on day 0 (Exp-1; high-nutrient prebloom conditions) and day 4 (Exp-2; low-nutrient postbloom conditions) of mesocosm water incubation during each cruise. For these experiments, 5-liter Whirl-Pak bags were filled with 3 liters of seawater, and nutrients were added establishing eight different enrichment treatments as follows: (i) control (C); (ii) inorganic nutrient (I); (iii) vitamin B_12_ (Sigma; V2876); (iv) vitamin B_1_ (Sigma; T4625); (v) inorganic nutrients and vitamin B_12_ (I+B_12_); (vi) inorganic nutrients and vitamin B_1_ (I+B_1_); (vii) vitamins B_12_ and B_1_ (B_12_+B_1_); and (viii) inorganic nutrients with vitamins B_12_ and B_1_ (I+B_12_+B_1_) (see Table 1 for details). Inorganic nutrients were added to prevent nutrient limitation from masking the responses to B vitamins. The nutrient concentrations of the additions were the same as those used in similar enrichment experiments in the sampling area (67). The amount of vitamin B_12_ and B_1_ experimentally added approximated maximum concentrations previously observed in coastal areas (36, 68, 69). The amount of B_12_ added was considerably higher than the maximum amount measured in the sampling area (27, 34). However, toxic effects of the added amount on phytoplankton could be disregarded, as vitamin B_12_ is typically added at much higher concentrations (370 to 400 nM) in phytoplankton culture media (45, 70, 71).

**TABLE 1:** List of enrichment treatments

Treatment no.	Treatment	Nutrient included	Concn
1	Control (C)	None	
2	Inorganic nutrients (I)	NO_3_^−^	5 μM
		NH_4_^+^	5 μM
		HPO_4_^2−^	1 μM
		SiO_4_^4−^	5 μM
3	Vitamin B_12_ (B_12_)	B_12_	100 pM
4	Vitamin B_1_ (B_1_)	B_1_	600 pM
5	B_12_+B_1_	B_12_	100 pM
		B_1_	600 pM
6	I+B_12_	NO_3_^−^	5 μM
		NH_4_^+^	1 μM
		HPO_4_^2−^	5 μM
		SiO_4_^4−^	5 μM
		B_12_	100 pM
7	I+B_1_	NO_3_^−^	5 μM
		NH_4_^+^	5 μM
		HPO_4_^2−^	1 μM
		SiO_4_^4−^	5 μM
		B_1_	600 pM
8	I+B_12_+B_1_	NO_3_^−^	5 μM
		NH_4_^+^	5 μM
		HPO_4_^2−^	1 μM
		SiO_4_^4−^	5 μM
		B_12_	100 pM
		B_1_	600 pM

Each treatment had three replicates, resulting in 24 Whirl-Pak bags per experiment. These experiments lasted 96 h, and *in situ* temperature was reached by submerging the bags in tanks filled with constantly circulating surface seawater.

In order to estimate the microbial biomass responses, Chl-*a* was measured daily in all treatments, and PB was measured after 96 h of incubation of addition experiments in February and after 72 h of experiments conducted in April and August. The time points for prokaryote biomass analyses were selected based on the highest Chl-*a* values, except for Exp-2 in February, where the maximum phytoplankton response occurred after 24 h. Microbial plankton community composition (determined by DNA sequencing) at the endpoint was analyzed in both experiments conducted in February, in Exp-2 in April, and in Exp-1 in August. Note that prokaryote community composition in the control treatment in the experiment Exp-1 in August was not available due to failed amplification. In order to explore changes in prokaryotic B_12_- and B_1_-related gene expression, RNA samples were taken at the endpoint of Exp-1 conducted in February. Due to budget constraints, the experiments for DNA and RNA analyses were selected from the observation of clear differential responses of phytoplankton and prokaryote biomass.

### Vitamin B_12_ concentration.

Mesocosm seawater (2 liters) was filtered through 0.2-μm Sterivex filter units under dim-light conditions and frozen at −20°C until further analysis. The methodology for concentration and detection of B_12_ was adapted from references 72–74 and is fully described in reference 34. Samples (1 liter) were preconcentrated using a solid-phase extraction (SPE) column (Econo-Pac chromatography columns; Bio-Rad) with 5 g of HF-Bondesil C_18_ resin (Agilent Technologies) at pH 6.5 and a rate of 1 ml min^−1^. Elution was performed with 12 ml of methanol (MeOH; liquid chromatography-mass spectrometry [LC-MS] grade), which was removed via evaporation with nitrogen in a Turbovap.

The analyses of dissolved B_12_ concentrations in seawater samples were carried out by liquid chromatography coupled to tandem mass spectrometry (LC-MS/MS). We report here the B_12_ forms that were analyzed, cyanocobalamin (CB_12_) and hydroxocobalamin (HB_12_). The total B_12_ concentration is therefore the sum of these two forms and should be considered a conservative estimate (27). Briefly, quantification of dissolved B_12_ (HB_12_ and CB_12_) was carried out using a high-performance liquid chromatography (HPLC) 1290 Infinity LC system (Agilent Technologies, Germany) coupled to an Agilent G6460A triple-quadrupole mass spectrometer equipped with an Agilent Jet Stream electrospray ionization (ESI) source (Agilent Technologies, Germany). The LC system used a C_18_ reverse-phase Agilent Zorbax SB-C_18_ rapid-resolution high-throughput column (2.1 by 50 mm [inside diameter], 1.8-μm particle size) with a 10-μl sample loop. The mobile phases consisted of LC-MS-grade water (solvent A) and methanol (solvent B), both buffered to pH 5.0 with 0.5% (vol/vol) of acetic acid (LC-MS grade). The chromatographic conditions consisted of an isocratic condition of 7% mobile phase B during 2 min, a gradient from 7% to 100% mobile phase B for the next 9 min, and an isocratic condition with 100% B for 2.5 min, returning to the initial conditions until completion of 15 min of run.

Limits of detection (LOD) were 0.04 pM for HB_12_ and 0.01 pM for CB_12_, while the limits of quantification (LOQ) were 0.05 and 0.025 pM for HB_12_ and CB_12_, respectively. The average B_12_ recovery after preconcentration and extraction of B-vitamin-spiked samples was 93%. Even though we set up the method for the detection of vitamin B_1_, we could not detect it in our samples, likely due to a low ambient concentration and the limited preconcentration volume (1 liter).

### Dissolved inorganic nitrogen.

Aliquots for inorganic nitrogen determinations (ammonium, nitrite, and nitrate) were collected in precleaned 50-ml polyethylene bottles (5% [vol/vol] HCl) employing contamination-free plastic gloves and immediately frozen at −20°C until analysis by standard colorimetric methods with an Alliance Futura segmented flow analyzer (75). The measurement error was 0.1 μM for nitrate, 0.02 μM for nitrite, and 0.05 μM for ammonium. DIN concentration was calculated as the sum of the ammonium, nitrite, and nitrate concentration.

### Dissolved organic matter.

Samples were collected in 250 ml acid-washed all-glass flasks and were gently filtered through acid-rinsed 0.2-μm filters (Pall Supor). Filtration was done in an acid-cleaned all-glass filtration device under low pressure of high-purity N_2_. Approximately 15 ml of the filtrate was collected in precombusted (450°C for 24 h) Wheaton amber glass vials of 20 ml stopped with acid-cleaned polytetrafluoroethylene (PTFE)-lined caps and immediately frozen at −20°C until analysis in the base laboratory. After defrosting, samples were acidified with 150 μl of 25% (vol/vol) H_3_PO_4_ and analyzed in a Shimadzu TOC-V analyzer coupled in series with a TNM-1 chemiluminescence detector. Reference materials provided by D. A. Hansell (University of Miami) were analyzed to check the accuracy of the instruments.

### Chlorophyll *a* concentration.

Chl-*a* concentration was measured as a phytoplankton biomass proxy. A volume of 300 ml of water was filtered through 0.2-μm-pore-size polycarbonate filters and frozen at −20°C until analysis. Chl-*a* from filters was extracted with 90% (vol/vol) acetone (HPLC grade) at 4°C overnight in dark conditions. Chl-*a* fluorescence was determined with a TD-700 Turner Designs fluorometer calibrated with pure Chl-*a* standard solution.

### Prokaryote biomass.

Samples (2 ml) for PB quantification were preserved with 1% (vol/vol) paraformaldehyde with 0.05% (vol/vol) glutaraldehyde, incubated for 20 min at room temperature, and stored at −80°C after being flash-frozen with liquid nitrogen. The abundance of heterotrophic prokaryotes was determined using a FACSCalibur flow cytometer (BD Biosciences, USA) equipped with a laser emitting at 488 nm. Samples were stained with SYBR green DNA fluorochrome prior to analysis, and prokaryote abundance was detected by their signature of side scatter (SSC) and green fluorescence as described by Gasol and Del Giorgio (76). The empirical calibration between light SSC and cell diameter described in reference 77 was used to estimate the biovolume (BV) of cells. BV was converted into biomass by using the allometric factor of Norland (78) (fg C cell^−1^ = 120 × BV^0.72^) for the coastal samples and using the open ocean conversion factor for the oceanic samples (fg C cell^−1^ = 350 × BV) (79).

### Microbial community composition.

A volume of 2 liters of water samples was sequentially filtered through 3-μm-pore-size polycarbonate filters (Whatman) and 0.22-μm-pore-size Sterivex-GP filter units (0.22 μm; EMD Millipore), immediately frozen in liquid nitrogen, and preserved at −80°C. In the case of addition experiments associated with the mesocosms, water was sampled from pooled experimental replicates, resulting in one sample per treatment and controls. DNA from biomass retained in the 3.0-μm and 0.2-μm filters was extracted using the PowerSoil DNA isolation kit (MoBio Laboratories Inc., CA, USA) and the PowerWater DNA isolation kit (MoBio Laboratories, Inc., CA, USA), respectively, according to the manufacturer’s instructions. DNA concentration was fluorometrically quantified with a Qubit 3.0 instrument and Qubit double-stranded-DNA (dsDNA) high-sensitivity assay kits (Invitrogen). Prokaryote community composition, mostly representing the free-living prokaryotes, was assessed by sequencing the V4 and V5 regions of the 16S rRNA gene (16S rRNA) of DNA from 0.2-μm Sterivex filters (3.0 μm prefiltered) by using the universal primers 515F and 926R (80). Eukaryote community composition from both 3-μm and 0.2-μm filters was assessed by sequencing the V4 region from the 18S rRNA gene (18S rRNA) using the primers TAReuk454FWD1 and TAReukREV3 (81). Amplified regions were sequenced with the Illumina MiSeq platform (paired-end reads; 2 × 300 bp) at the Research and Testing Laboratory (Lubbock, TX, USA) and subsequently denoised using the DADA2 pipeline (82). The SILVA reference database (83) was used for taxonomic assignment of 16S rRNA ASVs (amplicon sequence variants). PR2 (84) and the marine protist database from the BioMarks project (85) were used for the taxonomic assignment of 18S rRNA ASVs.

The ASV tables of prokaryotes and eukaryotes were subsampled to the lowest number of reads present in a sample, which was 2,080 and 1,286 (Fig. S1), for 16S rRNA and 18S rRNA, respectively. A total of 1,147 unique 16S rRNA ASVs of prokaryotes were identified. We combined data sets derived from the 0.2-μm and the 3-μm filters for eukaryote community analyses, since many ASVs of 18S rRNA were present in both size fractions. Reads from each filter size were normalized by the filter DNA yield, as explained in references 26 and 86, resulting in 2,293 unique 18S rRNA ASVs. The sequence abundances of the subsampled ASV tables were transformed using the centered log-ratio (clr) (87, 88), and this transformation does not admit zeros. Therefore, the zeros were replaced by the minimum value divided by 2, as described in reference 87.

### Metatranscriptomic analysis: bacterial community gene expression.

Water for metatranscriptomics was sampled from pooled experimental replicates, resulting in a data set covering two technical replicates per treatment of which one was sequenced per treatment. Approximately 2 liters of water was filtered through 3-μm-pore-size polycarbonate filters (Whatman) and Sterivex filter units (GP, 0.22 μm; EMD Millipore), preserved in 2 ml RNAlater (Qiagen), immediately flash frozen in liquid nitrogen, and stored at −80°C. The time between collecting the samples and storage never exceeded 20 min. Total RNA was extracted from the Sterivex filter using a protocol adapted from reference 89 with an RNeasy minikit (Qiagen) as described in reference 90. RNA was extracted by using RLT lysis buffer with β-mercaptoethanol (10 μl ml^−1^ RLT buffer) and mechanical zirconium bead beating (OPS Diagnostics) for 15 min at room temperature (Genie II; Scientific Industries), followed by centrifugation for 5 min at 3,260 × *g*. The RNA was diluted in an equal volume of 70% ethanol and purified by using the RNeasy minikit according to the manufacturer’s instructions. Total RNA was DNase treated using a Turbo DNA-free kit (Thermo Fisher Scientific) according to the manufacturer’s protocol and subsequently controlled for residual DNA by PCR with 16S rRNA primers (27F and 1492R) and visualization on an agarose gel. rRNA was depleted using a RiboMinus transcriptome isolation kit and RiboMinus concentration module (Thermo Fisher Scientific), and the remaining RNA was linearly amplified using the MessageAmp II-Bacteria RNA amplification kit (Thermo Fisher Scientific). Finally, cDNA was sent for sequencing at the National Genome Infrastructure, SciLifeLab Stockholm, on an Illumina HiSeq 2500 platform in rapid mode using HiSeq SBS kit v4 chemistry to obtain 2 × 126-bp paired-end reads.

The quality of individual paired-end reads was determined through FastQC (91) and MultiQC (92). Attached Illumina adapter sequences were removed with Cutadapt (93) version 1.13 and a set maximum error rate threshold of 0.1 (10%), and reads were trimmed with Sickle (94) version 1.33 in paired-end mode and Sanger quality values. Remaining rRNA sequences were bioinformatically filtered with ERNE (95) version 2.1.1 against an in-house database of stable RNA sequences from marine microbes. Subsequently, forward and reverse reads were merged with PEAR (96) version 0.9.10 with a minimum assembly length of 50 nucleotides (nt), a *P* value of 0.01, and a minimum overlap of 10 nt. The average fragment size was 308.73 ± 14.5 nt (*n* = 8). The proportion of joined reads was on average 94.5% ± 5.5% (*n* = 8). Merged reads were aligned with DIAMOND (97) version 0.8.26 against the NCBI RefSeq protein database (98). Subsequently, functional SEED classification (99) and taxonomic affiliation were assigned with MEGAN (100) version 6.7.3. Genes with a relative abundance of <1 cpm in all treatments were excluded from the study. Genes involved in the metabolism of B_12_ and B_1_ were analyzed and classified as “genes of synthesis,” i.e., genes involved in intracellular metabolic reactions, and “genes of uptake,” which encoded transporters required for the transport of exogenous molecules inside the cells (Tables S1 and S2). Results presented here indicate potential changes in relative transcription (in counts per million) of the different genes in the different addition treatments. No statistical analysis was performed for the comparisons, since only one replicate per treatment was available.

### Statistical analyses.

The effect of B-vitamin addition on Chl-*a* and PB was evaluated. Primary and secondary limitations by B vitamins were evaluated by applying paired *t* tests between the mean value in the B-vitamin treatment and the treatment with B-vitamin plus inorganic nutrients compared with control and inorganic nutrient treatments, respectively.

Principal-coordinate analysis (PCoA) of the Euclidean distance matrix of the prokaryote and eukaryote community composition at the endpoint of each treatment was used to visualize how microbial community composition changed after nutrient and/or B-vitamin additions. Analysis of similarity (ANOSIM; 999 permutations) was used to assess significant differences in both prokaryote and eukaryote community composition between experiments. In order to calculate species diversity of the microbial community, the Shannon-Weaver index (H′) was calculated for prokaryotes and eukaryotes with the function diversity from the R package vegan v2.4-2.

Differential abundance of ASVs between experimental treatments was analyzed based on a Wilcoxon rank sum test and Welch's *t* test running the ALDEx2 R package (101, 102). In order to identify prokaryote and eukaryote ASVs significantly and systematically responding to the B-vitamin additions throughout the year, only taxa present in all the control samples were included in the ALDEx2 analysis. To determine the effect of B-vitamin additions on populations, the effect size was calculated, which is the median of the ratio of the between group difference and the larger of the variances within groups. The Benjamini-Hochberg-Yekutieli procedure was used to account for multiple testing, and corrected values were expressed as false discovery rates (FDR) (103).

Nonmetric multidimensional scaling (NMDS) was used to analyze the similarity patterns in the bacterial gene expression (measured as counts per million) based on Bray-Curtis dissimilarity at the end of the addition experiment Exp-1 of February. In addition, an NMDS based on Bray-Curtis dissimilarity was performed to analyze the patterns in the expression of genes involved in B-vitamin metabolism between treatments.

### Data availability.

The data for this study have been deposited in the European Nucleotide Archive (ENA) at EMBL-EBI (https://www.ebi.ac.uk/ena) under accession numbers PRJEB36188 (16S rRNA sequences) and PRJEB36099 (18S rRNA sequences). The RNA data for this study have been deposited in the EMBL-EBI European Nucleotide Archive repository (https://www.ebi.ac.uk/ena) under the primary accession number PRJEB36712.
