# Supplementary material for: Microbial Plankton Community Structure and Function Responses to Vitamin B12 and B1 Amendments in an Upwelling System
Source: Appl Environ Microbiol. 2021 Oct 28;87(22):e01525-21. doi: 10.1128/AEM.01525-21 (PMC8552899; doi:10.1128/AEM.01525-21)
Supplement: Supplemental file 1 — Tables S1 to S4, Fig. S1 to S4. Download AEM.01525-21-s0001.pdf, PDF file, 0.5 MB [file aem.01525-21-s0001.pdf]

Supplementary Information

Table S1: Complete names and enzyme commission (EC) numbers of genes involved in vitamin B12 metabolism in bacteria. NA: data not available.

Table S2: Complete names and enzyme commission (EC) numbers of genes involved in vitamin B1 metabolism in bacteria. NA: data not available.

Table S3: Shannon diversity indices (*H*') of prokaryotes and eukaryotes in the selected experiments in February, April and August.

Table S4: Bacterial gene expression (measured as cpm) of overall metabolic functions (SEED categories) in the different treatments at the end of Exp-1 in February. cpm: counts per million.

Figure S1: Rarefaction curves showing the increase in richness with the increase in the number of 18S (top panel) or 16S (bottom panel) reads for all the samples sequenced within the ENVISION project.

Figure S2. Relative abundance of sequence reads assigned to the major taxonomic groups of prokaryotes and eukaryotes during the mesocosms conducted in February (A, B), April (C, D) and August (E, F) from the first day (d0) to the seventh day (d7). NA, not available due to failed amplification.

Figure S3: A non-metric multi-dimensional scaling (nMDS) showing the ordination of samples according to Bray-Curtis disimilarity between treatments (each colour) in the total bacterial gene expression at the end of Exp-1 in February. Colours correspond to the treatments.

Figure S4: Bacterial gene expression (measured as cpm) of the SEED categories showing differences in the relative abundance of transcripts (cpm) between treatments and control. cpm: counts per million. Colours correspond to the treatments.

Table S1

ID	Gene	EC	
**UroM**	Uroporphyrinogen-III methyltransferase	2.1.1.107	**B12 synthesis**
**CobA**	Cob(I)alamin adenosyltransferase	2.5.1.17	
**PduO**	Cob(I)alamin adenosyltransferase	2.5.1.17	
**CobT**	NicotiNDte-nucleotide-dimethylbenzimidazole phosphoribosyltransferase	2.4.2.21	
**CobNST**	CobN component of cobalt chelatase involved in B12 biosynthesis	NA	
**CobU/CobP**	AdenosylcobiNDmide-phosphate guanylyltransferase	2.7.7.62	
**CobD**	L-threonine 3-O-phosphate decarboxylase	4.1.1.81	
**CobB-CbiA**	Cobyrinic acid A,C-diamide synthase	NA	
**CbiK-SirCC**	Sirohydrochlorin cobaltochelatase	4.99.1.3	
**CobC**	Alpha-ribazole-5'-phosphate phosphatase	3.1.3.73	
**BluB**	5,6-dimethylbenzimidazole synthase	1.13.11.79	
**CobW**	CobW GTPase involved in cobalt insertion for B12 biosynthesis	NA	
**CbiF**	Cobalt-precorrin-4 C11-methyltransferase	2.1.1.133	
**CbiH**	Cobalt-precorrin-3b C17-methyltransferase	2.1.1.131	
**CbiD**	Cobalt-precorrin-6 synthase, aNDerobic	2.1.1.195	
**CbiT**	Cobalt-precorrin-6y C15-methyltransferase	2.1.1.196	
**CobS**	Cobalamin synthase	2.7.8.26	
**CbiC**	Cobalt-precorrin-8x methylmutase	5.4.1.2	
**CbiL**	Cobalt-precorrin-2 C20-methyltransferase	2.1.1.130	
**CobK**	Cobalt-precorrin-6x reductase	1.3.1.54	
**CbiG**	cobalt-precorrin 5A hydrolase	3.7.1.12	
**BtuB**	Outer membrane vitamin B12 receptor BtuB	NA	**B12 uptake**
**BtuF**	Vitamin B12 ABC transporter, B12-binding component BtuF	NA	
**BtuC**	Vitamin B12 ABC transporter, permease component BtuC	NA	
**BtuD**	Vitamin B12 ABC transporter, ATPase component BtuD	NA	
**BtuN**	Component of the B12 transporter BtuN	NA	

Table S2

**ID**	**Gene**	**EC**	
**TPK(ThiL)**	Thiamine-monophosphate kiNDse	2.7.4.16	**B1 synthesis**
**ThiC**	Hydroxymethylpyrimidine phosphate synthase	4.1.99.17	
**ThiF**	Sulfur carrier protein adenylyltransferase	NA	
**ThiG**	Thiazole biosynthesis protein	2.8.1.10	
**Rhod(ThiI)**	Rhodanese-like domain required for thiamine synthesis	NA	
**ThiE**	Thiamin-phosphate pyrophosphorylase	2.5.1.3	
**ThiI**	Thiamine biosynthesis protein	NA	
**ThiO**	Glycine oxidase	1.4.3.19	
**TPP-like**	Thiamin-phosphate pyrophosphorylase-like protein	NA	
**TPPK**	Thiamin pyrophosphokiNDse	2.7.6.2	
**ThiS**	Sulfur carrier protein	2.7.7.73	
**PnuT**	Predicted thiamin transporter PnuT	NA	**B1 uptake**
**Omr1**	Thiamin-regulated outer membrane receptor Omr1	NA	
**XT_19**	Xanthine/uracil/thiamine/ascorbate permease family protein	NA	
**ThiB**	Thiamin ABC transporter, substrate-binding component	NA	
**ThiY**	Hydroxymethylpyrimidine ABC transporter, substrate-binding component	NA	
**ThiQ**	Thiamin ABC transporter, ATPase component	NA	
**PT**	Cytosine/purine/uracil/thiamine/allantoin permease family protein	NA	
**ThiP**	Thiamin ABC transporter, transmembrane component	NA	
**ThiZ**	Hydroxymethylpyrimidine ABC transporter, ATPase component	NA	
**ThiX**	Hydroxymethylpyrimidine ABC transporter, transmembrane component	NA	

Table S3

Month	Experiment	Treatment	Prokaryote H'	Eukaryote H'
February	Exp-1	C	4.35	4.07
	Exp-1	B12	4.17	4.12
	Exp-1	B1	4.3	4.16
	Exp-1	B12B1	4.18	3.2
	Exp-1	I	4.01	3.95
	Exp-1	IB12	3.99	3.49
	Exp-1	IB1	4.16	4.24
	Exp-1	IB12B1	4.29	4.05
	Exp-2	C	4.27	4.62
	Exp-2	B12	3.83	4.56
	Exp-2	B1	4.02	4.35
	Exp-2	B12B1	4.37	4.74
	Exp-2	I	3.73	4.68
	Exp-2	IB12	3.88	4.37
	Exp-2	IB1	4.39	4.23
	Exp-2	IB12B1	4.26	4.34
April	Exp-2	C	4.6	4.14
	Exp-2	B12	4.52	4.29
	Exp-2	B1	4.46	4.45
	Exp-2	B12B1	4.43	4.13
	Exp-2	I	4.61	4.59
	Exp-2	IB12	4.67	4.35
	Exp-2	IB1	4.26	4.33
	Exp-2	IB12B1	4.54	4.28
August	Exp-1	C	NA	4.8
	Exp-1	B12	4.73	4.85
	Exp-1	B1	4.55	4.2
	Exp-1	B12B1	4.72	4.82
	Exp-1	I	4.45	4.87
	Exp-1	IB12	4.85	4.61
	Exp-1	IB1	4.86	5.08
	Exp-1	IB12B1	4.83	NA

Table S4

SEED	C	B12	B1	B12B1	I	IB12	IB1	IB12B1
**Protein Metabolism**	323111	289974	312893	316018	264239	372012	388104	333979
**Carbohydrates**	142430	151487	139485	137052	164915	136539	133112	164902
**Amino Acids**	138766	131892	124372	126434	150495	117659	126087	136432
**Virulence**	137690	148797	153138	142979	136073	154031	151472	147696
**Cofactors/Vitamins**	113033	121218	111753	116752	133638	103262	106688	118893
**Unclassified**	107323	104314	97592	101865	111635	102209	98728	114407
**RNA Metabolism**	93224	92875	96003	92733	93364	104147	104201	93991
**Respiration**	92247	89286	96124	104323	82025	103214	98746	96191
**Membrane Transport**	67164	57599	55390	51244	49002	44452	36582	45370
**Miscellaneous**	58200	55139	53702	59563	58959	49651	58563	56274
**Phosphorus Metabolism**	46376	42097	43856	49363	29130	23152	22290	13859
**Stress Response**	41712	52683	48273	47894	54446	39997	38698	46540
**Mitochondrial electron transport**	40673	35983	39703	45515	32963	51006	45049	44253
**Nitrogen Metabolism**	40560	37463	43977	44948	42129	41147	46002	38857
**Nucleosides and Nucleotides**	36854	31827	31426	37148	33321	27498	33049	30964
**Fatty Acids, Lipids**	35000	39386	34619	34815	54124	45992	38678	54444
**Cell Wall and Capsule**	34946	35130	34181	35683	43199	30326	31963	33126
**Phages and Plasmids**	33352	43101	41742	46186	27751	23510	24857	22876
**Iron metabolism**	32100	22134	22523	21757	18655	18920	14957	19906
**DNA Metabolism**	29564	30587	27006	28387	43763	23941	23982	29446
**Motility and Chemotaxis**	25529	61336	52907	34263	56077	41961	31496	52463
**Aromatic Compounds**	24151	16123	14694	15086	22225	16462	16679	21123
**Cell Division and Cell Cycle**	19882	20089	19189	21578	20875	17677	19375	19710
**Regulation and Cell signaling**	19614	30147	25942	22141	27560	20979	21011	25580
**Metabolite damage**	18825	17186	16818	19305	20046	14807	16647	16165
**Sulfur Metabolism**	18341	16201	14816	16002	20264	16940	19017	20147
**Predictions by plant-prokaryote**	17191	18129	18145	18650	19261	20414	21142	16770
**Nucleotide sugars**	6681	6114	6264	6798	5272	3762	3777	4741
**Photosynthesis**	5879	12355	10539	12416	5738	3957	5141	7416
**Potassium metabolism**	4678	6164	5371	5179	8349	4784	4556	5405
**Secondary Metabolism**	2610	3515	3351	3518	4480	3001	2839	3564
**Autotrophy**	2594	3096	3656	3742	4409	4075	5335	3858
**Plant Glucosinolates**	2477	2675	2313	2179	3524	3031	2470	2965
**Central metabolism**	1876	2908	2815	3057	3783	2529	2486	3160
**Transcriptional regulation**	1452	1845	1631	1737	2346	1067	1098	1465
**Stationary Phase Response**	1306	3525	3316	1490	2519	1801	966	2638

**Dormancy and Sporulation**	1002	1211	1115	1314	1912	955	899	1170
**Plastidial electron transport**	484	1048	858	904	440	361	419	609
**Polyamines**	461	338	267	284	430	332	361	352

Figure S1

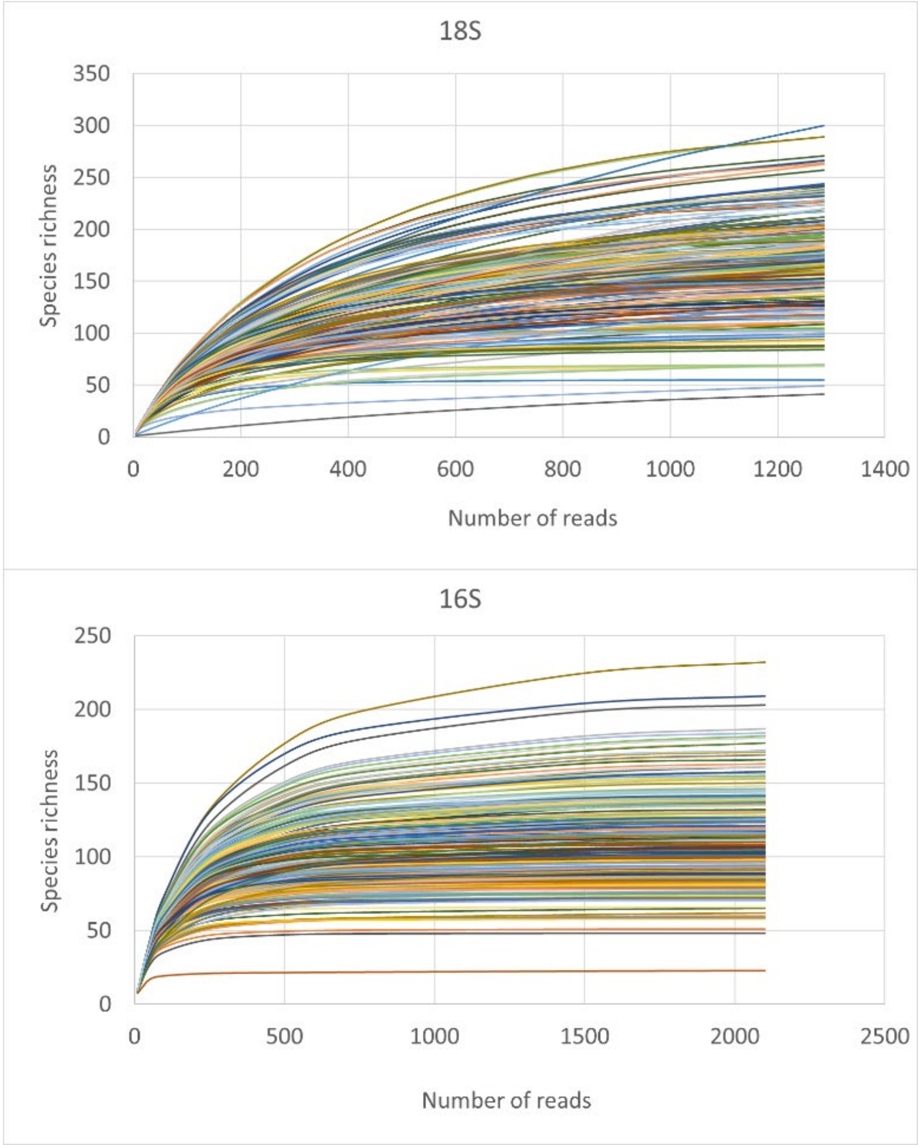
18S
Species richness
Number of reads
0
50
100
150
200
250
300
350
0
200
400
600
800
1000
1200
1400
16S
Species richness
Number of reads
0
50
100
150
200
250
0
500
1000
1500
2000
2500

Figure S2

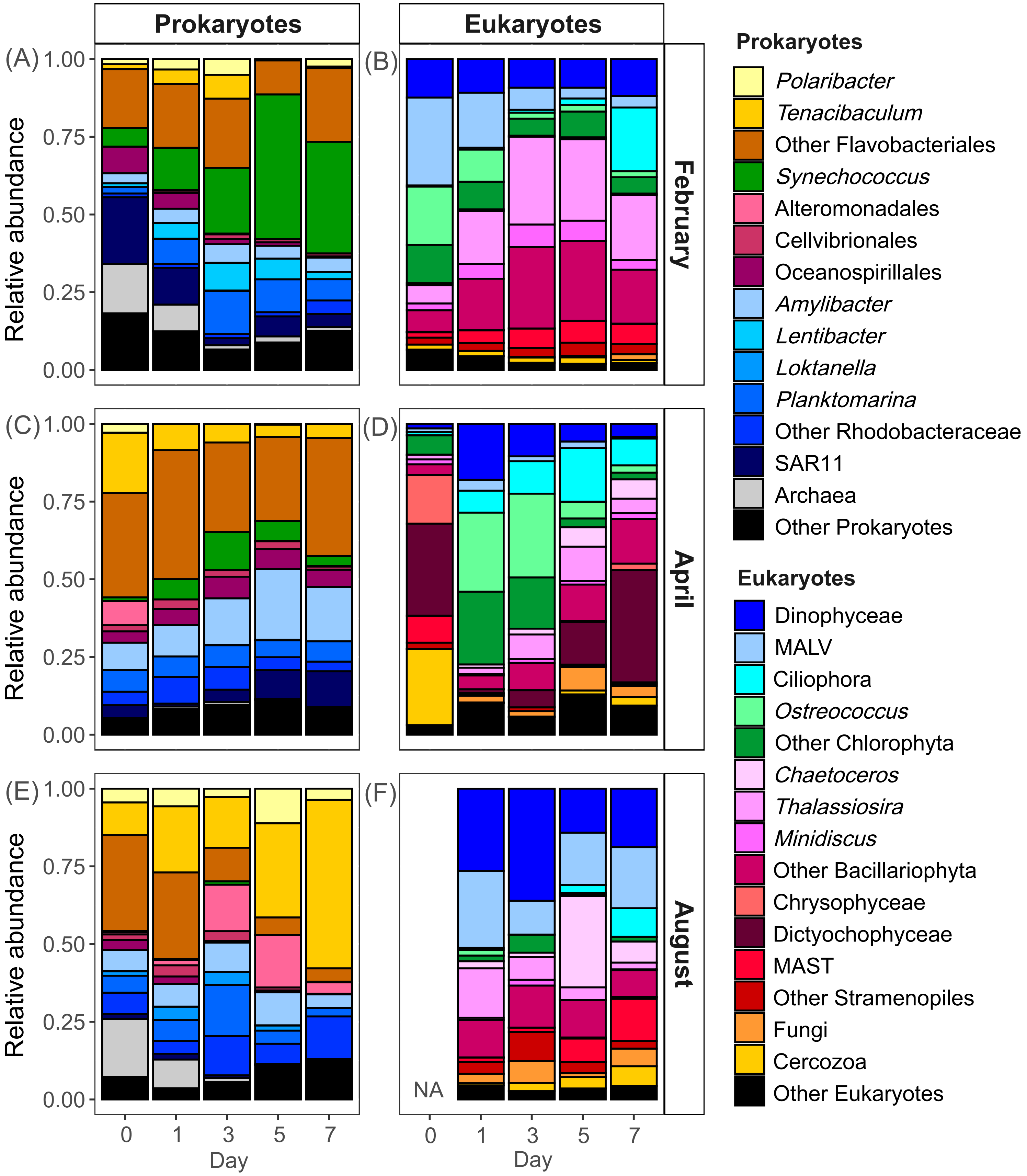
Prokaryotes
Eukaryotes
(A)
(B)
(C)
(D)
(E)
(F)
Relative abundance
1.00
0.75
0.50
0.25
0.00
February
April
August
NA
0
1
3
5
7
Day
Prokaryotes
Polaribacter
Tenacibaculum
Other Flavobacteriales
Synechococcus
Alteromonadales
Cellvibrionales
Oceanospirillales
Amylibacter
Lentibacter
Loktanella
Planktomarina
Other Rhodobacteraceae
SAR11
Archaea
Other Prokaryotes
Eukaryotes
Dinophyceae
MALV
Ciliophora
Ostreococcus
Other Chlorophyta
Chaetoceros
Thalassiosira
Minidiscus
Other Bacillariophyta
Chrysophyceae
Dictyochophyceae
MAST
Other Stramenopiles
Fungi
Cercozoa
Other Eukaryotes

Figure S3

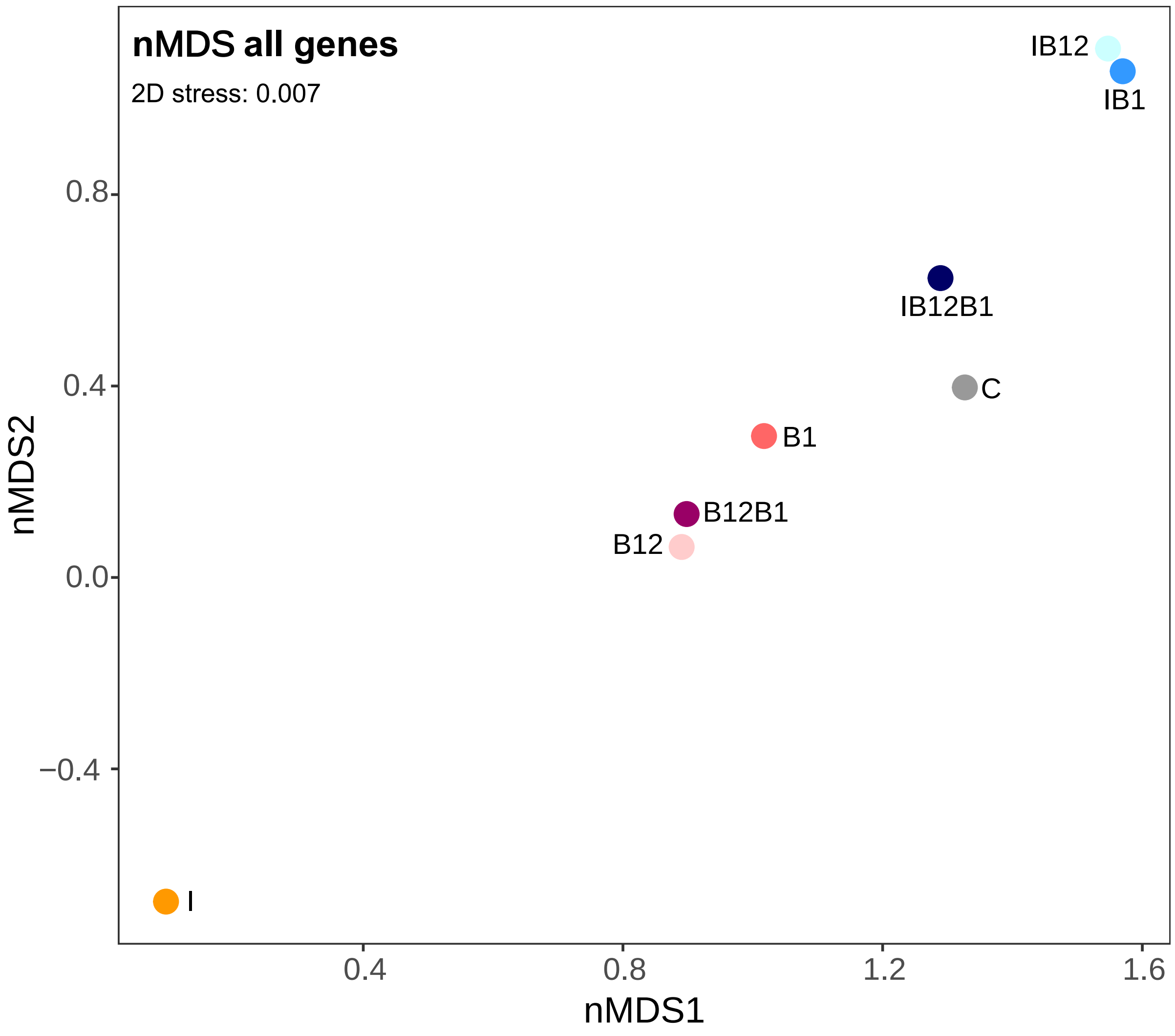

nMDS all genes
2D stress: 0.007
IB12
IB1
IB12B1
C
B1
B12B1
B12
I
0.8
0.4
0.0
−0.4
0.4
0.8
1.2
1.6
nMDS2
nMDS1

Figure S4

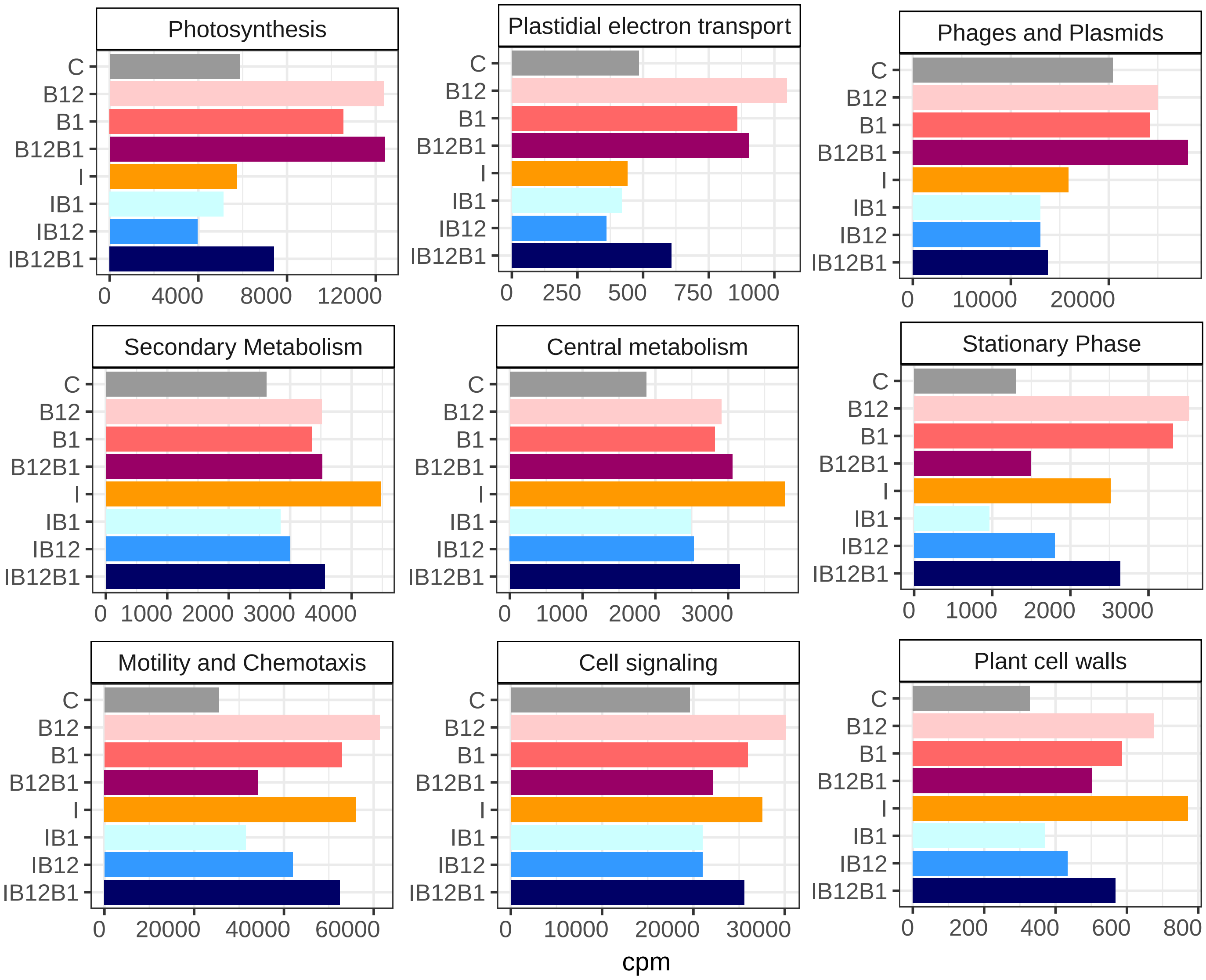
Photosynthesis
Plastidial electron transport
Phages and Plasmids
Secondary Metabolism
Central metabolism
Stationary Phase
Motility and Chemotaxis
Cell signaling
Plant cell walls
C
B12
B1
B12B1
I
IB1
IB12
IB12B1
cpm
